# Skeletal muscle metabolism and contraction performance regulation by teneurin C-terminal-associated peptide-1

**DOI:** 10.3389/fphys.2022.1031264

**Published:** 2022-11-29

**Authors:** David W. Hogg, Andrea L. Reid, Thomas L. Dodsworth, Yani Chen, Ross M. Reid, Mei Xu, Mia Husic, Peggy R. Biga, Andrew Slee, Leslie T. Buck, Dalia Barsyte-Lovejoy, Marius Locke, David A. Lovejoy

**Affiliations:** ^1^ Department of Cell and Systems Biology, University of Toronto, Toronto, ON, Canada; ^2^ Department of Biology, University of Alabama, Birmingham, AL, United States; ^3^ Protagenic Therapeutics, Inc., Lexington, MA, United States; ^4^ Structural Genomics Consortium, University of Toronto, Toronto, ON, Canada; ^5^ Department of Kinesiology, University of Toronto, Toronto, ON, Canada

**Keywords:** energy, mitochondria, CRISPR, calcium, ATP, evolution, teneurin, latrophilin

## Abstract

Skeletal muscle regulation is responsible for voluntary muscular movement in vertebrates. The genes of two essential proteins, teneurins and latrophilins (LPHN), evolving in ancestors of multicellular animals form a ligand-receptor pair, and are now shown to be required for skeletal muscle function. Teneurins possess a bioactive peptide, termed the teneurin C-terminal associated peptide (TCAP) that interacts with the LPHNs to regulate skeletal muscle contractility strength and fatigue by an insulin-independent glucose importation mechanism in rats. CRISPR-based knockouts and siRNA-associated knockdowns of LPHN-1 and-3 in the C2C12 mouse skeletal cell line shows that TCAP stimulates an LPHN-dependent cytosolic Ca^2+^ signal transduction cascade to increase energy metabolism and enhance skeletal muscle function *via* increases in type-1 oxidative fiber formation and reduce the fatigue response. Thus, the teneurin/TCAP-LPHN system is presented as a novel mechanism that regulates the energy requirements and performance of skeletal muscle.

## 1 Introduction

Skeletal muscle is critical for all voluntary behaviors and is derived from the earliest contractile proteins present in the ancestral single-celled heterotrophs. Enhanced contractile strength and efficient energy metabolism among these primitive skeletal muscle cells were, therefore, required for both locomotion and feeding (Ni et al., 2012; Ramulu et al., 2012). We have postulated that essential intercellular signaling systems, originating phylogenetically early, conferred a selective advantage upon these basal heterotrophs by linking sensory and motor functions with cell metabolism (Lovejoy and Hogg, 2020). Numerous studies have indicated that the teneurins and their receptors, the latrophilins (LPHN) are part of an ancient regulatory system that modulates cell adhesion and metabolism. The introduction of the teneurin and LPHN genes into multicellular animals occurred *via* lateral gene transfer from prokaryotes into a single-celled ancestor of metazoans (King et al., 2003; King et al., 2008; Strotmann et al., 2011; Zhang et al., 2012; Chand et al., 2013a; Tucker, 2013). Thus, the teneurins and LPHNs were evolutionarily poised to play a seminal role in the development and coordination of cell-to-cell communication, adhesion and metabolic activities.

Teneurins are essential for development and maintenance of the central nervous system (CNS) (Baumgartner et al., 1994; Levine et al., 1994; Rubin et al., 1999; Tucker and Chiquet-Ehrismann, 2006; Kenzelmann et al., 2008; Young and Leamey, 2009; Hong et al., 2012; Mosca et al., 2012; Li et al., 2018). Comprising a family of four paralogous proteins in vertebrates, the teneurins possess several functional domains that confer specialized actions on their extracellular and intracellular regions (Minet et al., 1999; Oohashi et al., 1999; Minet and Chiquet-Ehrismann, 2000). As type-II proteins, their carboxyl terminus is displaced extracellularly. The distal region contains a β-barrel structure unique to metazoans, but is similar to that found in prokaryotic Tc-toxins (Wang et al., 2005; Lovejoy et al., 2006; Aravind et al., 2012; Zhang et al., 2012; Jackson et al., 2018; Li et al., 2018). Associated with the extracellular domain lies an extended amino-acid chain termed the ‘teneurin C-terminal associated peptide’ (TCAP) (Wang et al., 2005; Lovejoy et al., 2006; Jackson et al., 2018). The TCAPs possess primary structure similarity to the Secretin superfamily of peptides that include, not only secretin paralogs such as vasoactive intestinal peptide (VIP), growth hormone-releasing hormone (GHRH), glucagon and pituitary adenylate cyclase activating peptide (PACAP), but also the calcitonin and corticotropin-releasing factor (CRF) families. One of the distinguishing aspects of this peptide superfamily is their role in the coordination of sensory, motor and energy metabolism (Sekar and Chow, 2013; Lovejoy and Hogg, 2020; Michalec et al., 2020).

The LPHNs are G-protein-coupled receptors (GPCR) belonging to the Latrophilin Adhesion GPCR family (ADGRL) and are cognate receptors of the teneurins in vertebrates (Fredricksson et al., 2003; Fredriksson and Schiöth, 2005; Araç and Li, 2019). LPHNs have three distinct paralogous forms (LPHNs-1-3) and can bind to the C-terminal region of the teneurins, which include the TCAP region. For example, Teneurin-2 and LPHN-1 binds with nanomolar affinity at the lectin-domain of LPHN-1 (Silva et al., 2011). A splice variant of C-terminal domain of teneurin-2, also termed ‘LPHN1-associated synaptic surface organizer’ (Lasso), binds to LPHN with, likewise, high affinity in neurons. Moreover, transgenic over-expression of both TCAP-1 and the hormone-binding domain (HBD) of LPHN-1 results in co-precipitation of both transgenic proteins indicating an interaction between TCAP-1 and LPHN-1 (Husic et al., 2019). Recent structural studies of the teneurins indicate that the TCAP region may be auto-catalytically cleaved from the teneurins after interaction with the LPHNs (Jackson et al., 2018; Li et al., 2018), or could be the result of a separate teneurin splice variant resulting in the mRNA expression of the terminal exon that includes the TCAP sequence (Chand et al., 2013b; Reid et al., 2021).

Regardless of the mechanism of TCAP release, the expected TCAP mature peptide, based on its genomic sequence, has distinct biological properties. Synthetic TCAP-1 regulates cytoskeletal elements and energy metabolism in neurons critical for neuroplastic modulation in the CNS. In rats, TCAP-1 modifies dendritic arborization and spine density in the hippocampus (Tan et al., 2008; Tan et al., 2011), findings that were corroborated in primary cultures of rat embryonic hippocampal tissues that exhibited increased filopodia, neurite and axon development (Al Chawaf et al., 2007a; Chand et al., 2012; Chand et al., 2013b). Thus, these actions indicate a role of TCAP-1 in CNS energy metabolism. Moreover, subcutaneous administration of TCAP-1 into rats increases brain glucose uptake as assessed by functional positron emission tomography (fPET). These observations were corroborated by the expected decreased serum glucose and insulin levels in rats, and in cell culture studies showing that TCAP-1 stimulates glucose uptake by increased glucose transporter transit to the membrane and subsequently increases in ATP turnover providing increased energy for the neurons (Hogg et al., 2018).

However, given the evolutionary history of the teneurins, it is plausible that the teneurins, LPHNs, and TCAP could also play a role in the regulation of skeletal muscle. Skeletal muscle is one of the most important sites of glucose metabolism and is responsible for 40% of glucose-associated energy requirements (Hogg et al., 2018) and 80% of glucose uptake under insulin-stimulated conditions (Richter and Hargraves, 2013). Muscle function and metabolism are intrinsically linked, as evidenced by metabolic syndromes that result in poor muscle function and degradation. A key example of this is demonstrated in patients with type-II diabetes where patients have reduced skeletal muscle function in the grip force test compared to non-diabetic patients (Sayer et al., 2005; Santos et al., 2008).

Based on these previous findings, we investigated the role of TCAP-1 on skeletal muscle function for the first time. We demonstrate that skeletal muscle possesses the critical elements of teneurin-LPHN interaction, and show that TCAP-1 regulates skeletal muscle contractile kinetics *in vivo* in rats. These studies are supplemented by *in vitro* studies, using the mouse skeletal cell line, C2C12, to show that TCAP-1 regulates intracellular skeletal calcium (Ca^2+^) flux similar to that reported in neurons (Hogg et al., 2018; Lovejoy and Hogg, 2020). Moreover, like neurons, the TCAP-1-mediated Ca^+2^ response leads to increased glucose metabolism and mitochondrial activation, but results in skeletal muscle fiber regulation. We posit that the teneurin-LPHN interaction is required for skeletal muscle physiology and regulates skeletal muscle performance.

## 2 Methods

### 2.1 Peptide synthesis and solubilization

**FIGURE 1:**
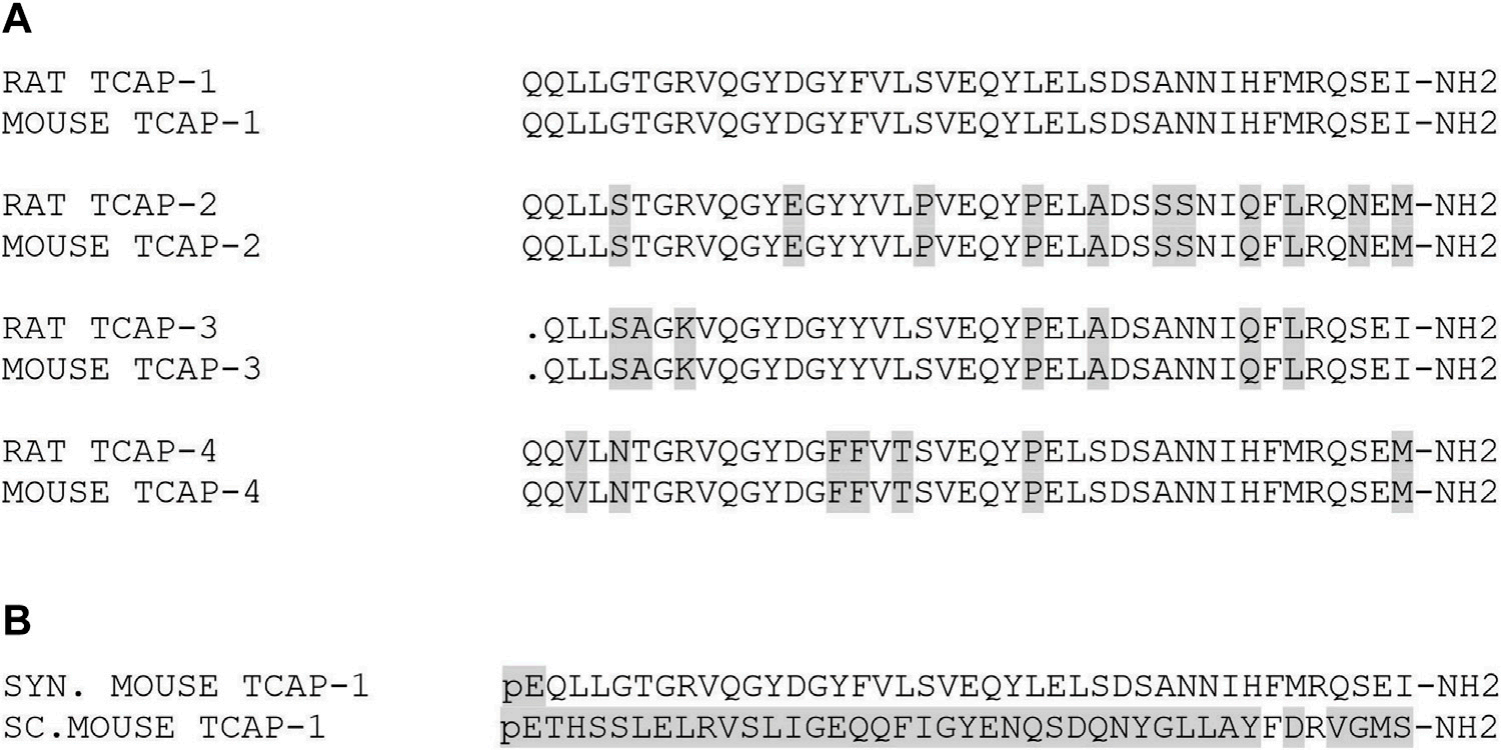
Primary structures of rat and mouse TCAP peptides. **(A)**. Comparison of the amino acid sequences of mouse and rat TCAPs. **(B)**. Primary structure of the peptides used in this study. Grey boxed regions indicates regions of identity relative to the rat/mouse TCAP isoforms. Note: pE refers to glutamine residues that have been modified to pyroglutamyl residues and–NH_2_ indicates where the carboxy terminus has been expected to be modified to an amidated product. In addition, the synthetic peptides, **(B)** have been prepared with both modifications based on their genomic sequences.

Both peptides; rat/mouse TCAP-1 and scrambled (sc)TCAP-1 (Figure 1), were synthesized commercially by AmbioPharm, Inc. and prepared as an acetylated salt at 95% purity. Peptides were solubilized in saline after alkalization as previously described (Al Chawaf et al., 2007a) then diluted into the required media for *in vitro* or *in vivo* studies (see below). The primary structure of all 4 rat and mouse TCAPs are identical to each other’s paralog (Figure 1A) and possess a 73–83% sequence identity among the overall orthologous sequences, although most of these changes reflect homologous and conservative substitutions. For this reason, TCAP-1 was used in both rat and mouse preparations. Synthetic rat/mouse TCAP-1 was prepared with an initial N-terminal pyroglutamic acid to inhibit N-terminal-directed peptidases, and a C-terminal amidated-residue as expected based on the genomic sequence (Wang et al., 2005; Al Chawaf et al., 2007a). As a control peptide, we have utilized a scrambled (sc) amino acid sequence version of rat/mouse TCAP-1 where each residue, with the exception of the initial pyroglutamyl residue (pE), was randomized in its placement within the peptide (Figure 1B). This scTCAP-1 has been used in previous studies to establish an additional level of controls to ensure that TCAP-1 is not affecting non-specific (e.g. oligopeptide transporters; non-target receptors) actions. The vehicle included scTCAP solubilized in 0.9% saline or cell culture medium, unless otherwise stated.

### 2.2 Animals

Male adult Sprague-Dawley (SD) rats (Charles River, Canada) were used for the short-term and long-term muscle function studies. The metabolic and endocrine studies of TCAP-1 on rats were approved by the University of Toronto Animal Care Committee (UACC) under the auspices of the Canadian Council of Animal Care. Male adult Wistar rats (∼250 g) (Charles River, United States) were used for the functional positron emission tomography (fPET) studies performed by Molecular Imaging, Inc. (Ann Arbor, MI, United States) and approved by the American Association of Animal Laboratory Care (Hogg et al., 2018). In both sets of studies, animals were weighed weekly and monitored for any signs of distress or illness (e.g., loss of hair, extreme weight loss, abnormal behaviors). Animals were monitored daily by animal care personnel under the supervision of the facility veterinarian. Had any animals exhibited signs of distress, they would have been removed from the study and humanely euthanized using CO_2_ exposure. However, no animals showed overt indications of stress and, subsequently, all animals were utilized for these studies.

### 2.3 *In Vivo* studies

The short-term application studies of TCAP-1 utilized 16 male adult SD rats (250g) that were acclimated for 1 week (w) on a 12:12 light-dark (LD) cycle. For 5 days (d) daily, the animals were treated with either vehicle or TCAP-1 (10 nmol/kg) by subcutaneous (SC) injection in the intrascapular region. Animals were tested for muscle function by electrical muscle stimulation (see below) 3 days after the last treatment under anesthesia of 5% isoflurane when animals were immediately euthanized afterward using CO_2_. For the long-term study, 20 adult male SD rats (350g), acclimated for 1w on a 12:12 LD cycle and were treated with either vehicle or intrascapular TCAP-1 (SC; 25 nmol/kg) for 3 months (m) (1 injection/week). Muscle function by electrical muscle stimulation was tested 2w after the last treatment. Animals were immediately euthanized after electrical muscle stimulation studies (see below).

#### 2.3.1 PCR-based mRNA expression of teneurin and LPHN

RNA was extracted from tibialis anterior (TA) muscles using TRIzol (Thermo Scientific, Waltham, MA, United States) following the manufacturer’s instructions. The PCR reaction mix included 5 μL cDNA, 2 μL forward primer and 2 μL reverse primer (Invitrogen; Table 1), 14.2 μL water (Sigma, Oakville, ON), 3 μL 10x Taq Buffer with KCl (Thermo Scientific), 1.8 μL MgCl_2_ (Thermo Scientific), 1 μL deoxynucleotide Solution Mix and 0.5 μL Taq DNA Polymerase (New England Biolabs). The reactions were incubated in an Eppendorf Mastercycler Gradient Thermal Cycler for 7 m at 95°C; followed by 35 cycles of 60 s at 95 °C, 90s at 67°C, and 35s at 72°C; and then held at 4°C. cDNA samples were resolved on a 3% agarose gel at 100 V for 1.5 h and visualized using a Bio-Rad ChemiDoc MP System with 0.5s exposure.

**TABLE 1:** Primers used for *in vitro* and *in vitro* RT-PCR analyses. Forward and reverse primer pairs for the four teneurins, TCAPs, three latrophilins (ADGRL), and β-actin control are indicated.

Gene	Forward primer (5′-3′)	Reverse primer (5′-3′)	Expected band size
Teneurin-1	CAC​AGT​CAG​CGG​CGT​TAC​ATC​TTT​GAG	GAA​TCC​GTC​ATG​CAT​CAG​GTG​TAT​TGT	342 bp
Teneurin-2	ATC​CTG​AAC​TCG​CCG​TCC​TCC​TTA	CTC​CAG​GTT​CTG​AGT​GGA​CAC​GGC	405 bp
Teneurin-3	GTG​AGT​ACC​GTT​GAT​GTC​AAA​GAT​G	AGT​GGA​ATA​CCC​GGT​GGG​GAA​GCA​C	427 bp
Teneurin-4	ATC​GAC​CAA​TTC​CTG​CTG​AGC​AAG	CCA​GAG​AGG​CAT​CCC​GGT​AGA​GTC	567 bp
TCAP-1	ACG​TCA​GTG​TTG​AAT​GGG​AGG​ACT​A	CCT​CCT​GCC​TAT​TTC​ACT​CTG​TCT​CAT	351 bp
TCAP-2	GAC​AAG​ATG​CAC​TAC​AGC​ATC​GAG	CCA​TCT​CAT​TCT​GTC​TTA​AGA​ACT​GG	496 bp
TCAP-3	CAA​CAA​CGC​CTT​CTA​CCT​GGA​GAA​C	CGA​TCT​CAC​TTT​GTC​GCA​AGA​ACT	506 bp
TCAP-4	TTT​GCC​TCC​AGT​GGT​TCC​ATC​TT	TGG​ATA​TTG​TTG​GCG​CTG​TCT​GAC	602 bp
ADGRL1	AGC​CAG​AGG​ACT​TGA​CTC​A	TTC​TAG​GCC​TCA​GAG​CTA​CAT	249 bp
ADGRL2	TGGAGCAAAAAGTC	TTCAAAACAGC	203 bp
ADGRL3	TGA​GCA​ACT​GTG​TGC​AAA​TT	TAA​CCA​CCA​GCC​ACA​CCA​T	327 bp
β-actin	CAG​CCA​TGT​ACG​TAG​CCA​TCC​A	ATG​TCA​CGC​ACG​ATT​TCC​CTC​T	247 bp

#### 2.3.2 Histological studies

TA muscle was excised, then flash-frozen in liquid nitrogen-cooled isopentane. The tissue was cryo-sectioned at 10 μm at −20°C and transferred to cover-slides and fixed using ice-cold methanol. Immunohistochemical (IHC) studies of the β-dystroglycan (β-DG) and teneurins followed previously established methods using commercially available monoclonal antibodies (Al Chawaf et al., 2007a; Chand et al., 2012; Chand et al., 2013b; Husic et al., 2019). Following blocking for 1 h with 10% normal goat serum (NGS: Cell Signaling, Inc.), the primary antibodies against β-dystroglycan (β-DG; 1:500 dilution, Santa Cruz Biotechnology), teneurin-3 (1:1200 dilution; Abnova) or latrophilin (1:1000 dilution: Santa Cruz Biotechnology) diluted in 1% NGS, were added and incubated overnight (ON) at 4°C. After 3x phosphate-buffered saline (PBS) washing, the cells were treated with the secondary antibodies, Alexa 594, goat anti-rabbit (1:400 dilution; Invitrogen) or Alexa 488 donkey anti-mouse (1:400 dilution, Invitrogen) and incubated for 1 h at room temperature (RT) in the dark. Coverslips were attached, and the tissue imaged using confocal microscopy (Leica TCS-SP8) at ×400 magnification. For fluorescence analyzes of protein expression, ImageJ software was used to measure arbitrary fluorescent units (AFU), where changes of AFU are proportional to protein expression changes. A total of five slides were quantified with eight regions of interest (ROI) investigated. ROI was defined as regions with multiple cell interactions free of artifacts.

#### 2.3.3 functional Positronic Emission Tomography (fPET)

Male Wistar rats were treated with either vehicle or TCAP-1 (10 nmol/kg) by intrascapular SC injection. After 3 days of treatment, 1 mCi of [^18^F]-2-deoxyglucose (^18^F-DG) radiotracer (IBA Molecular) was administered intravenously (IV) under 5% isoflurane anesthesia. fPET scans were performed using a Siemens Inveon microPET small animal PET scanner using the protocol as previously described (Hogg et al., 2018). Briefly, body temperature was maintained with a thermostat-regulated recirculating water-heated pad. Static emission data was acquired for 20 m. The PET list mode data was converted to 2-dimensional (2D) sinograms, corrected for random coincidences, and normalized for scanner uniformity. The PET image analysis was performed using the Amira 5.5.0 analysis software package. For whole body ROIs (regions of high ^18^F-DG uptake), a low threshold was set to delineate specific signals in the whole body while eliminating background. The total PET counts were calculated from all voxels within the segmented volumes of interest. These images were then compiled into 3-dimensional (3D) projections, thus allowing for accurate analyzes of muscle tissue. Fluorescence of the mean pixel was calibrated to volume of muscle being analyzed (mean pixel fluorescence/mm^3^).

#### 2.3.4 NADH staining and analysis

TA muscles from the treated SD rats were cryo-sectioned at 10 μm thickness as described above. Cryo-sections were washed 2x with PBS, then 0.2% NBT solution in PBS (containing 0.1% NADH; Sigma, Oakville, ON, Canada) was added and incubated for 30 m at 37 °C. Slides were washed 2x in PBS before mounted, imaged at ×100 magnification and analyzed on ImageJ software for pixel density, where darker pixels represent higher levels of NADH. Expression of NADH was analyzed based on a minimum of 100 fibers per tissue, with a minimum of 3-4 tissues analyzed for each group.

#### 2.3.5 Muscle function and integrity testing by electrical muscle stimulation

The electrical muscle stimulation protocol was followed as described previously (Holwerda and Locke, 2014) with minor modifications. Briefly, both TCAP-1 or non-treated animals were anesthetized with 5% isofluorane in 1L/min O_2_, and subsequently positioned into the testing apparatus. A 25g needle was inserted through the soft tissue of the knee in order to ensure a stable position. The foot was placed on a lever attached to a servomotor and taped in position. Electrodes were placed below the skin but adjacent to the TA muscle. Dynamic Muscle Control (DMC; Version five; Aurora Scientific) software was used for electrical stimulation and analyses. The correct voltages for peak tetanic tension was established by increasing voltage by 1V increments until optimal tetanus twitch was achieved. Subsequently, the test began with a single tetanus and single twitch protocol to establish the baseline followed by a 6 m fatigue protocol (8V, 200 Hz, 300 ms). After the termination of the protocol, tetanic and twitch tensions were recorded at 0, 1, and 5 m. Animals were immediately euthanized after recovery measurements were recorded.

#### 2.3.6 Quantitative reverse-transcription polymerase chain reaction

TA muscle mRNA and cDNA were prepared as previously described (see above). The cDNA from all samples was used to prepare pools to establish standard curves of each gene. The cDNA pool or cDNA samples were mixed with MasterMix containing SYBR select. The reactions were loaded in a 384-well PCR plate and run in a BioRAD qRT thermal cycler for 2 m at 50°C, 7 m at 95°C; followed by 39 cycles of 60s at 95°C, 90s at 67°C, and 35s at 72°C. Melting curves were established by a step-wise gradient from 60 to 90 °C. The myosin heavy chain (MHC) isoforms, MHCI, MHCIIa, MHCIIx and MHCIIb were analyzed by real-time PCR using the mRNA and cDNA prepared above (see Table 2).

**TABLE 2:** Rat MHC isoforms and β-actin control primers used for qRT-PCR. Forward and reverse primer pairs are indicated for the four MHC isoforms.

Gene	Forward primer (5′-3′)	Reverse primer (5′-3′)
MHCI	GAA​TGG​CAA​GAC​GGT​GAC​TGT	GGA​AGC​GTA​CCT​CTC​CTT​GAG​A
MHCIIa	ATG​ACA​ACT​CCT​CTC​GCT​TTG	TTA​AGC​TGG​AAA​GTG​ACC​CGG
MHCIIb	GAA​CAC​GAA​GCG​TGT​CAT​CCA	AGG​TTT​CGA​TAT​CTG​CGG​AGG
MHCIIx	CCA​ATG​AGA​CTA​AGA​CGC​CTG​G	GCT​ATC​GAT​GAA​TTG​TCC​CTC​G
β-actin	AGCCATGTACGTAGCCA	CTC​TCA​GCT​GTG​GTG​GTG​AA

### 2.4 *In Vitro* studies

#### 2.4.1 Culture, immunocytochemistry and mRNA expression studies of the C2C12 cell line

The immortalized murine skeletal muscle cell line, C2C12, was used for all *in vitro* studies. Cells were maintained at 60–70% confluency with Dulbecco’s Modified Eagle Medium (DMEM) supplemented with 20% fetal bovine serum (FBS) and a penicillin/streptomycin antibiotic combination (Invitrogen, Burlington, ON, Canada). To induce differentiation, the media was changed to DMEM supplemented with 10% horse serum (HS) with the penicillin/streptomycin antibiotic combination, and the cells were allowed to differentiate for 6 days (media replaced every 24 h). For treatment, cells were serum-starved for 3 h and then provided with either vehicle or TCAP-1 (100 nM). The TCAP immunocytochemistry (ICC) was performed using a polyclonal antiserum raised against TCAP-1 and previously characterized (Chand et al., 2013b). Briefly, cells were plated on poly-D-lysine-coated coverslips (BD Biosciences) and cultured overnight (ON) to 60% confluency in DMEM with 10% FBS. The coverslips were rinsed with PBS and fixed in 4% PFA and permeabilized with 0.3% Triton X-100 (Fisher Scientific). After blocking with 10% NGS (Vector Laboratories) for 1 h, the coverslips were incubated ON with the TCAP-1 TNR308 primary antiserum (1:1000 dilution) (Chand et al., 2013b). After incubation and washing, the cells were treated with the secondary antibodies indicated above. Where appropriate, the cells were washed then counterstained with DAPI, and mounted with DABCO. Fluoroceinisothiocyanate (FITC) was covalently linked to the K_37_ residue of mouse TCAP-1 peptide using the EZ-Label FITC protein labelling kit (Pierce Biotechnology) as we have used in the past (Al Chawaf et al., 2007a; Chand et al., 2012; Chand et al., 2013b). The identification of teneurin, TCAP and LPHN cDNAs in C2C12 cells were performed with mRNA extracted from differentiated mouse C2C12 cells using the method described above using the primer sequences indicated in Table 1.

#### 2.4.2 Live-cell calcium imaging in C2C12 myotubules

The C2C12 skeletal cells were grown and differentiated on poly-D-lysine-coated 25 mm round No. 1 glass coverslips (Warner Instruments, Hamden, CT, United States). Changes in intracellular Ca^2+^ were assessed using the membrane-permeable fluorescent indicator Fluorophore-4 (Fluo-4) (Invitrogen, Burlington, ON, Canada). Cells were loaded with Fluo-4 by incubating coverslips in DMEM containing 10 μM Fluo-4 for 30 m (37°C) followed by a 15 m wash in Locke’s Buffer (305–310 mOsmol at 22°C). In experiments assessing changes in intracellular Ca^2+^, coverslips were placed in a flow-through bath chamber (RC-40HP, Warner Instruments, Hamden, CT, United States) of an inverted microscope (Axio Observer Z1, Zeiss, Toronto, ON, Canada) equipped with a ×40 oil immersion objective. Cells were continuously bulk-perfused with Locke’s Buffer *via* a gravity drip perfusion system at a rate of 2–3 ml/min at RT. Changes in Fluo-4 fluorescence were imaged using a green fluorescent protein (GFP) filter set (Semrock, Rochester, NY, United States) and a X-Cite 120 fluorescence illumination system (Excelitas Technologies, Mississauga, ON, Canada), controlled by Volocity 4.0 imaging software (Quorum Technologies Inc., Guelph, ON, Canada). Fluorescence emissions were detected with an Orca-ER Hamamatsu B/W CCD digital camera (Hamamatsu, Middlesex, NJ, United States). Fluo-4 was excited with a wavelength of 480 nm for 100 ms every 3–5 s and fluorescence emission was measured at wavelength of 516 nm.

#### 2.4.3 Caffeine stimulation experiments

Caffeine treatment of the C2C12 cells was used to establish that the cells were viable. Caffeine (4 mM; Sigma-Aldrich, Oakville, ON, Canada) was applied to C2C12 myotubules to stimulate Ca^2+^ release from the sarcoplasmic reticulum (SR). Cells were either pre-treated with TCAP-1 (100 nM) or vehicle for 1 h before stimulation with caffeine. Using Velocity 4.0 imaging software, ROIs were taken from cytosolic regions within the myotubules (*n* = 4 coverslips, 4-5 ROIs per coverslip).

#### 2.4.4 GLUT4 immunocytochemical studies

C2C12 cells were differentiated as described above. After 3 h of serum-starvation, myotubules were treated either with vehicle, TCAP-1 (100 nM) or insulin (100 nM) for 15 or 30 m. Cells were then fixed using 4% PFA and subsequently blocked with 10% NGS for 1 h at RT. The GLUT4 primary antibody, diluted in 1% NGS, was added to the cells and incubated at 4 °C OT. Following 4x PBS washes, the secondary antibody (diluted in 1% NGS) was added and incubated 1 h at RT. The coverslips were mounted using DAPI-containing Vectashield. Slides were imaged on a confocal microscope with a ×40 oil objective. The images were analyzed using ImageJ, where myotubules were selected as ROIs and were analyzed for red pixel intensity values, representing ir-GLUT4 levels, and normalized to area size (*n* = 3-4 coverslips per treatment, 7-8 myotubules per coverslip). For the IP3R inhibitor, 2-aminoethoxydiphenyl borate (2-APB; Sigma Aldrich, Oakville, ON, Canada) experiments, 2-APB (100 µM) was applied for 4 m before the start of treatment with either sham (Locke’s buffer) or TCAP-1 (100 nM), containing 2-APB for continuous blocking of IP3R.

#### 2.4.5 Radioactive glucose uptake

The ^3^H-2-deoxyglucose uptake protocol was followed as previously described with minor modifications (Maher, 1995; Uemura and Greenlee, 2006). At day-6 post-plating, C2C12 myotubules were washed 2x with Locke’s Buffer without serum and glucose. The culture was incubated in the Locke’s Buffer for 1 h at 37 °C followed by exposure to 100 nM insulin, 100 nM TCAP-1, 100 nM SC-TCAP-1, or saline. ^3^H-2-deoxyglucose (0.5 μCi/ml) was added to the culture 5 m before termination of treatment exposure. Uptake of ^3^H-2-deoxyglucose was stopped immediately after 5 m with 3x washes of ice-cold 0.9% saline solution. The cells were digested with 1 ml of 0.05 M NaOH at 0, 30, 45, and 60 m after treatment. Radioactivity of the cell lysates was measured using a beta (β) -liquid scintillation counter (Beckman Coulter), and recorded in counts per minute (CPM).

#### 2.4.6 Intracellular ATP and NADH assays

ATP assays were conducted using Promega ATP Assay kits (Wisconsin, United States) following the manufacturer’s instructions. Briefly, C2C12 cells were seeded at 10,000 cells/well in 96-well plates. The following day, cells were treated with either vehicle or TCAP-1 (100 nM) and lysed at 0, 15, 30, and 60 m after treatment. Ultra-Glo recombinant luciferase (Promega, Wisconsin, United States) was added to the media to determine ATP levels. Fluorescence from blank wells was subtracted from all samples to account for background signal noise. As the fluorescence signal naturally decays over the course of the experiment, TCAP-1-treated cells were compared to the vehicle-treated cells for each time point (*n* = 8). The resazurin-resorufin-based NADH assays (Abcam) for the wild-type (WT) C2C12 and the CRISPR-associated knockout (KO) C2C12 cells, C2C12 myoblasts were seeded at 10,000 cells/well into a white clear-bottom 96-well plate and grown for 12–18 h. The resazurin sodium salt (0.025 mg/well from 10X stock in PBS) was added to each well and mixed for 1 m. Fluorescence was measured using the Tecan Infinite M1000 Pro plate reader, where each well was excited at 530 nm and emission was captured at 590 nm. Measurements were taken every 10 m from 0 to 30 m to establish a pre-treatment baseline. Then, vehicle (distilled water), TCAP-1 (100 nM) or FCCP (10 uM; all from 10X stocks in PBS) was added to each well and mixed for 1 m. Measurements were taken every 30 m for 150 m. To analyze, background fluorescence measurements from wells containing no cells were subtracted from sample fluorescence measurements. Relative fluorescence was calculated at each time point by normalizing to the background-subtracted fluorescence (in arbitrary fluorescence units, AFUs) at time 0, just prior to treatment. Each well represents *n* = 1.

#### 2.4.7 Diacylglycerol and inositol triphosphate (IP3) assays

The protocols provided by commercial DAG and IP3 assays (MyBiosource, San Diego, California, United States) were followed. To determine the downstream Ca^2+^ response, six replicates of C2C12 cells were prepared using the TCAP-1 treatment protocol described above then treated with either vehicle, the IP3R antagonist, 2-APB, or the phospholipase C inhibitor, U73122. Cell lysates were added to a microELISA plate coated with purified mouse DAG or IP3 antibodies. Subsequently, 3,3′,5,5′-tetramethylbenzidine (TMB) solution was added to detect the HRP-conjugates as color changes. Finally, sulfuric acid (0.01N) was added to terminate the reaction. The absorbance change was measured at 450 nm by spectrophotometry (SpectraMax Plus, NH, United States). For the IP3R inhibitor, 2-APB (100 µM) was applied before the start of treatment with either sham (Locke’s Buffer with scTCAP-1) or TCAP-1 (100 nM). For live-cell fluorescence experiments, C2C12 cells were differentiated and intracellular Ca^2+^ flux was assessed *via* Fluo-4 *via* a flow-through bath chamber of an inverted microscope. Cells were quantified with a GFP filter set at 480 nm with the fluorescence emission measured at 516 nm.

#### 2.4.8 Mitochondrial Ca^2+^ accumulation and membrane potential measurement in C2C12 myotubules

Changes in mitochondrial (MT) Ca^2+^ levels were assessed using fluorescent indicator, Rhodamine-2 (Rhod-2). C2C12 myotubules were loaded with Rhod-2 by incubating coverslips in DMEM containing 4 µM Rhod-2 (from a 1 mM stock solution in DMSO with 20% pluronic; Invitrogen-Pluronic™ F-127) for 30 m at 22 °C. Cells were washed once for 30 m at 37 °C in Locke’s Buffer Cells then acclimated for 15 m at 22°C. To assess changes in MT Ca^2+^ levels, cells were continuously perfused in a flow-through chamber as indicated previously. Changes in Rhod-2 fluorescence was imaged using a TRITC filter set (Semrock, Rochester, NY, United States) and an X-Cite 120 fluorescence illumination system (Quorum Technologies, Inc. Guelph ON, Canada). Emissions were detected using an Orca-ER Hamamatsu BW CCD digital camera as described above. Rhod-2 was excited at 552 nm every 100 ms and measured at 577 nm. Multiple ROI were taken from the nuclear regions of the myotubules (*n* = 5, 5-7 ROIs per coverslip). Changes in MT membrane potential were assessed using rhodamine-123 (Rhod-123)-based fluorescence. C2C12 myotubules were prepared by incubating coverslips in DMEM containing 5 μM Rhod-123 for 30 m (37 °C) followed by a 15 m wash in Locke’s Buffer. Changes in Rhod-123 fluorescence were imaged using the green GFP filter set using the same experimental configuration as previously described. Rhod-123 was excited with a wavelength of 480 nm for 100 ms every 5s and fluorescence emission was measured at 516 nm.

#### 2.4.9 SiRNA knockdowns and CRISPR knockouts of LPHN-1 and -3

**FIGURE 2:**
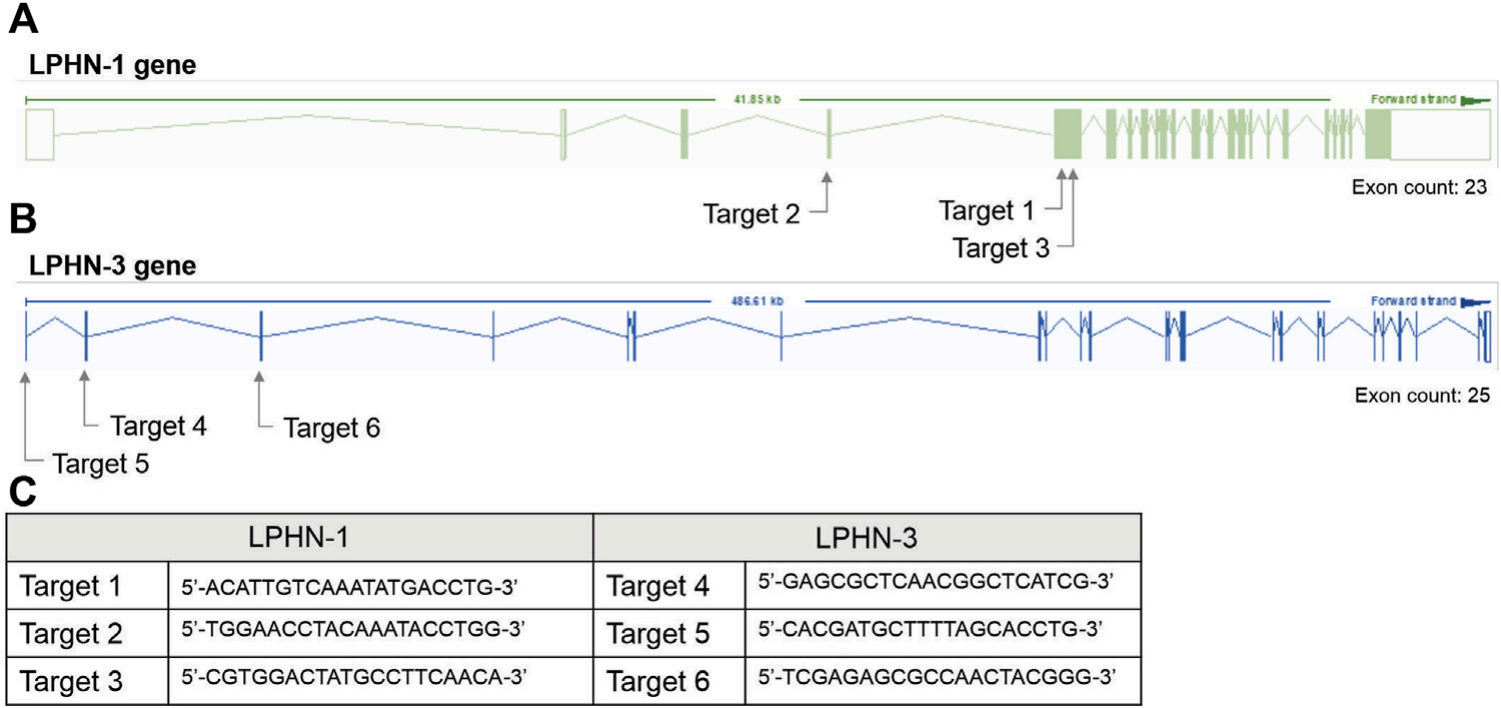
CRISPR-based targets for the mouse LPHN-1 and -3 genomic sequences. **(A)**. Schematics of LPHN-1 and -3 genomic organization. The oligonucleotides targeting these regions are indicated as arrows. **(B)**. Sequence of the oligonucleotides used in the CRISPR knockdowns as indicated in “A” above. **(C)** Sequence of oligonucleotides indicated in A and B above.

For the siRNA KD studies, transfection with siRNA oligonucleotides was performed after 4 days of C2C12 differentiation using Dharmacon SmartPOOL (Horizon Inc. Canada) siRNA for LPHN1 (L-061299-00-0005), LPHN3 (L-040779-00-0005) and a randomized non-targeting control (D-001810-10-05). Dharmacon SmartPOOL siRNAs targeted against LPHN-1 and-3, glyceraldehyde-3-phosphate (GAPD) and a non-targeting control were re-suspended in 1x siRNA buffer from 20 µM stocks. The stocks were diluted in serum-free and antibiotic-free DMEM to 250 nM. A 7.5 µL aliquot of Mirus TranslT-X2 (Mirus Bio LLC) transfection reagent was diluted in 200 µL serum- and antibiotic-free DMEM and incubated at RT for 30 m. The mixture was added to C2C12 cells (see above) with a final siRNA concentration of 25 nM. The cells were differentiated in siRNA-containing media for 2 days for a total of 6 days of differentiation before use in the experiments. For CRISPR studies, single-guided RNA (sgRNA) constructs were designed to target the mouse LPHN-1 and -3 gene at three locations (see Figure 2). C2C12 cells were transfected with sgRNA constructs (Figure 2C) and a Cas9 plasmid, generating heterogenous pools of transfected cells. The CRISPR/Cas-transfected C2C12 cells (either heterogenous pools or clones) were trypsinized and pelleted for DNA extraction. Genomic DNA was extracted using Lucigen QuickExtract DNA extraction solution (Biosearch Technologies, Inc.) according to the manufacturer’s direction. The LPHN-1 and -3 genes were amplified by PCR and digested by T7 endonuclease using the EnGen Mutation Detection Kit (New England Biolabs) according to directions in combination with the custom primers that flank the appropriate CRISPR-targeting regions (Figure 2C). The fragments were identified as previously described above. Clones that showed low or no wild-type (WT) PCR amplicon were screened for LPHN1 expression by qRT-PCR. Selected clones showing significantly reduced LPHN1 mRNA expression by qRT-PCR were termed ‘LPHN1 E5U KO’ and ‘LPHN1 E5D KO’ based on the exon position of mutated site and were used for further study (Figure 2). The activity of the clones were determined by TCAP-1-induced cytosolic Ca^2+^ flux, peroxisome proliferator-activated receptor γ coactivator 1α (PGC-1α) qRT-PCR-based expression, and NADH turnover using the methods described above.

### 2.5 Statistical analyses

The data graph data are represented as mean ± SEM. All data were analyzed by Student’s t-test or one or two-way ANOVA, as described within each figure caption. Tukey’s post-hoc test and Sidak’s post-hoc test were used to determine significance in one-way and two-way ANOVA analyzes, respectively, as indicated. An *a priori* hypothesis of *p* < 0.05 was used as a threshold for statistical significance. GraphPad Prism 7-8 was used to analyze each statistical test.

## 3 Results

The primary structure of rat and mouse TCAP-1 possesses a high degree of homology among the other three paralogs (Figure 1A). Because the primary structure of rat and mouse TCAP-1 is identical, it was used for all studies, as well as a proxy for the other TCAP isoforms. The first *in vivo* studies established that the teneurins, TCAP and LPHNs could be expressed in rat skeletal muscle and subsequently, the physiological role of TCAP-1 in the rat skeletal muscle was examined. In the second part of the study, we have used mouse C2C12 cells as a model to understand the molecular and cellular action on TCAP-1 in skeletal muscle function.

### 3.1 *In Vivo* rat studies

#### 3.1.1 Molecular expression

**FIGURE 3:**
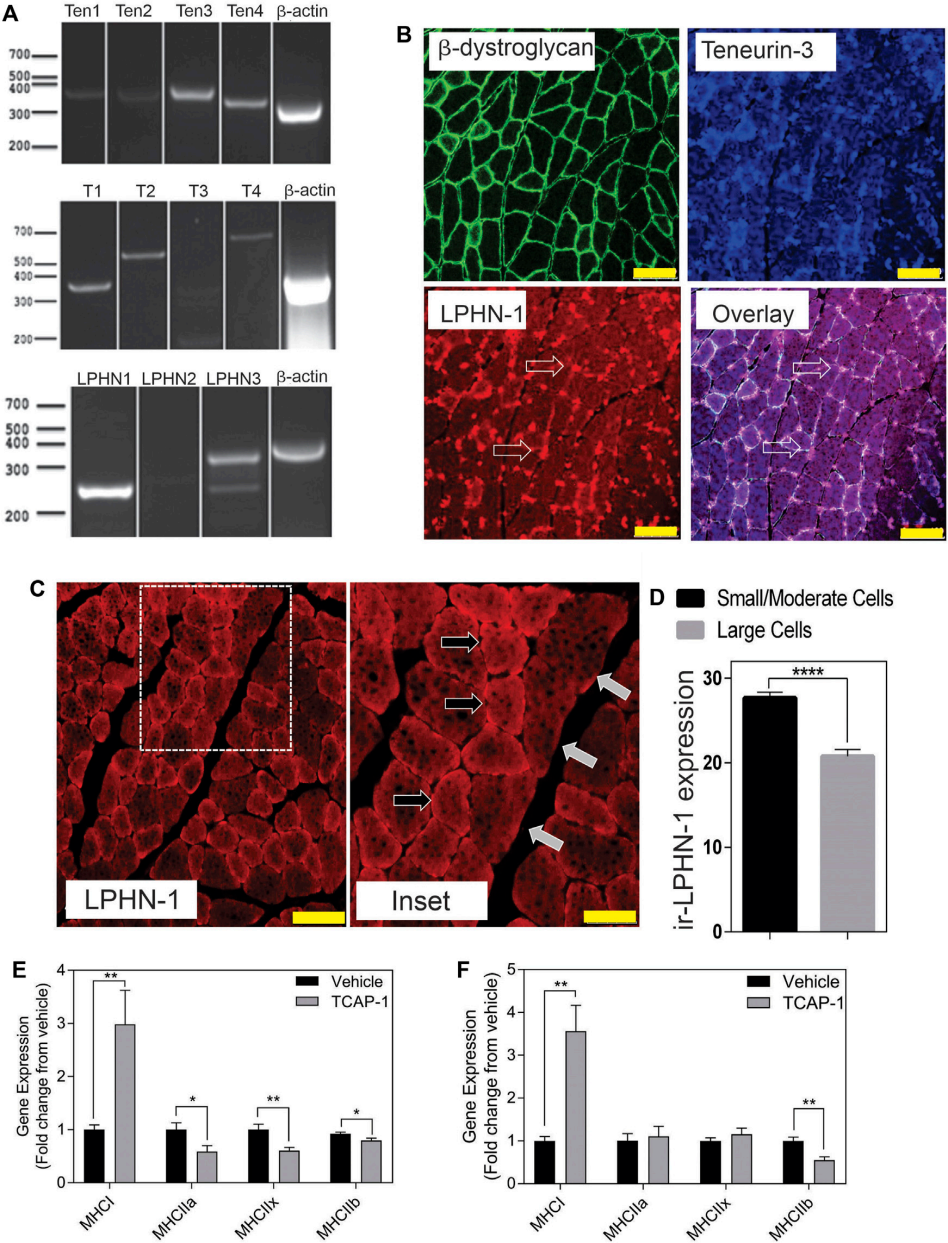
Expression of the teneurin/TCAP-LPHN immunoreactivity (ir) in rat skeletal muscle: **(A)**. PCR expression of teneurins, TCAP and LPHNs in rat TA muscle. **(B)**. Immunological expression of β-dystroglycan, teneurin and LPHN in rat skeletal muscle. Arrows indicate nodes of aggregation (Scale bar: 100 µM). **(C)**. Enhanced examination of ir-LPHN regions in TA muscle cells. Left panel scale bar indicates 100 μM, whereas the right panel scale bar indicates 50 µM. Black arrows indicate cells with high LPHN-1 labelling, whereas white arrows indicate cells of low LPHN-1 labelling. **(D)**. Quantification of ir-LPHN-1 as a function of muscle cell diameter (size) as shown in “C” (Student’s *t*-Test *p* < 0.0001; ****). **(E)**. Changes in fiber type in short-term TCAP-1 administration (*t*-test indicated for each pair). **(F)**. Changes in fiber-type over long-term TCAP-1 administration (*t*-test indicated for each pair).

In rat TA muscle mRNA extracts, all 4 teneurin mRNAs were identified based on the PCR primers indicated in Table 1. Teneurins-3 and-4 showed the strongest response, although both teneurins-1 and -2 were present, albiet weakly expressed. In contrast, TCAP-1 and -2 showed a strong signal relative to that indicated by teneurins-1 and -2 whereas TCAP-4 showed a signal consistent with teneurin-4. Although these studies were not quantitative, they do establish that both teneurins and TCAP paralogs are present in rat skeletal muscle. However, importantly, both LPHN-1 and -3 cDNA bands were also strongly expressed, although there was no evidence of LPHN-2 in this preparation (Figure 3A). To corroborate these cDNA studies, IHC expression was performed in rat TA muscle tissue. Initially, β-dystroglycan (DG) labelling was used to establish the sarcolemmic boundary of the cells, as previous studies indicated a relationship between DG and TCAP signaling (Chand et al., 2013b). Further, IHC co-localization labeling of teneurin-1,-3 -and LPHN-1 was utilized to determine the cellular anatomical relationship between the teneurins and LPHNs. Immunoreactive (ir) teneurin-1 labelling did not show a strong signal, consistent with the RT-PCR data indicated above, however the ir-teneurin-3 showed a response indicating specific concentrations of both ir-dystroglycans and ir-teneurins in the sarcolemma (Figure 3B). Importantly, ir-LPHN-1 labelling of these tissues showed co-localization with the ir-DG along with ir-teneurin and ir-TCAP labelling consistent with the PCR studies indicated in Figure 3A. The variation among the PCR-based mRNA and ir-teneurin -1 and -3 expression was expected due to affinity differences among the antibodies and primers (see Discussion). Moreover, these studies establish a clear relationship between TCAP, teneurins and LPHNs in rat skeletal muscle (Figure 3B). Morphological differences, with respect to cross-section diameter between the vehicle- and TCAP-1-treated animals could be discerned. Thus, TCAP-1 administration induced a 25% increase (*p* < 0.001) between the number of small and intermediate cells relative to the untreated vehicle rats (Figures 3C,D). Because small and intermediate fibers are typically oxidative muscle fibers, these observations suggested that TCAP could stimulate glucose uptake in skeletal muscle. To corroborate these findings, the expression of myosin heavy chain (MHC) was evaluated in the TA muscle of both short-term and long-term TCAP-treated animals. In both cases, there was a significant (*p* < 0.01) 3- to 3.5-fold increase in the expression of the MHCI fibers in the TCAP-1-treated animals compared to the non-treated vehicle, although significant (*p* < 0.05) differences were also observed among MHCIIa, MHCIIx and MHCIIb expression among the treated and untreated animals (Figures 3E,F).

#### 3.1.2 Glucose uptake and energy substrate studies

**FIGURE 4:**
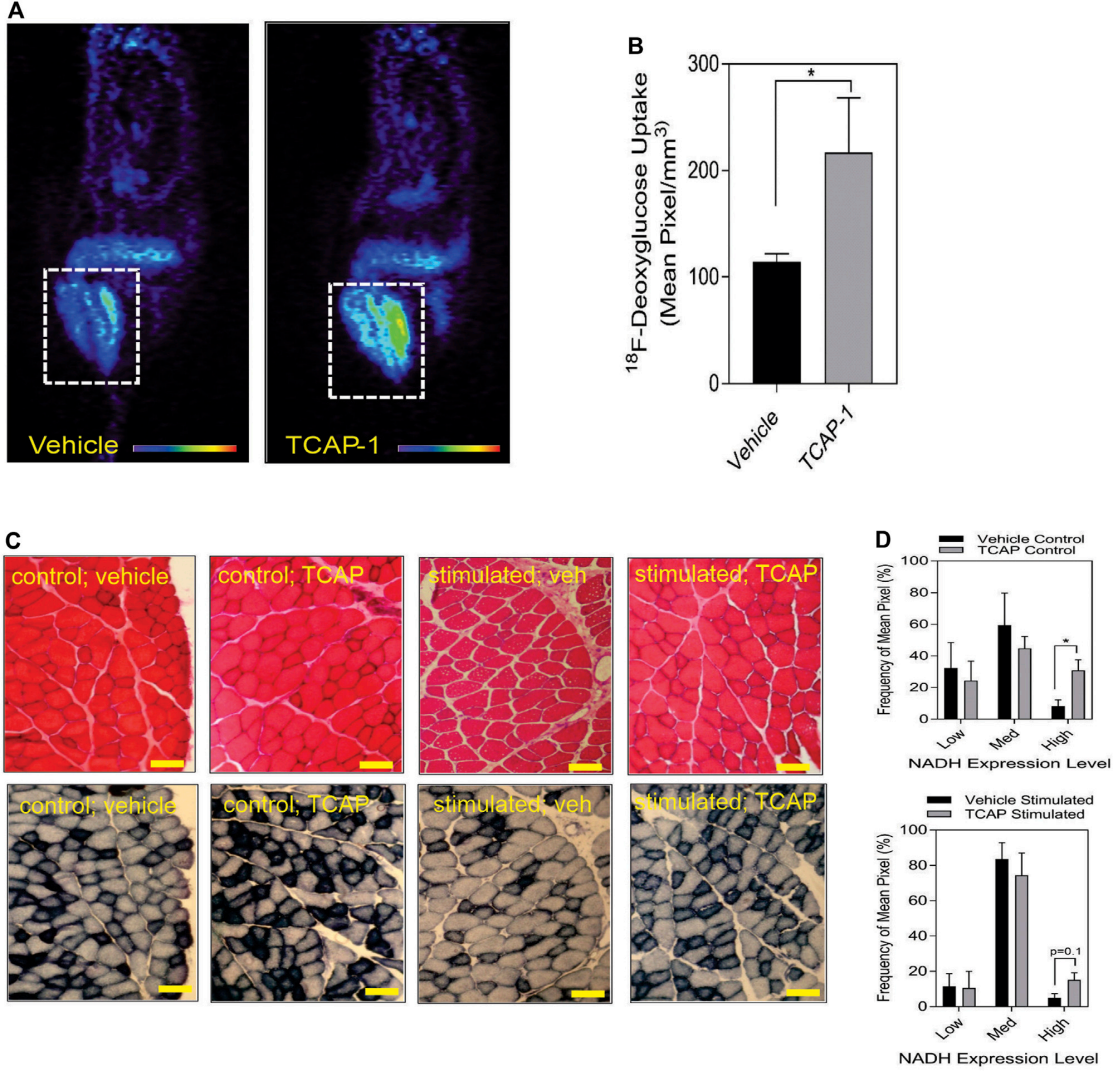
Glucose uptake and metabolism in rat hind-limb and TA muscle. **(A)** functional Positronic Emission Tomography of rat hind limb showing increase of ^18^F-deoxyglucose (^18^F-DG) uptake after 3 days from TCAP-1 administration. **(B)**. Quantification of the ^18^F-DG uptake in hindlimb after 3 days. (*n* = 5; student’s *t*-test; *p* < 0.05). **(C).** Stimulation of NADH in TA muscle after administration of TCAP-1. Above panel, (hematoxylin and eosin stain), bottom panel, NADH activity (shown as black regions). Scale bar indicates 100 µM. **(D)**. Quantification of the NADH-labelled cells shown in “C”. * *p* < 0.05; **p < 0.01; ***p < 0.001; ****p < 0.0001. Mean ± SEM indicated *n* = 4.

Taken together, these studies indicated that TCAP-1 may increase glucose transport into skeletal muscle. Therefore, TCAP-1-induced glucose uptake into the hind-limb was measured directly by fPET. Thus, using ^18^F-deoxyglucose (FDG), a single dose of TCAP-1-treatment induced FDG uptake in the hind-limb muscle by 2-fold (*p* < 0.05) (Figures 4A,B) after 3 days of treatment relative to vehicle treatment in contrast to the scTCAP-1 treatment. These data corroborated our supposition that TCAP-1 acted, in part, to increase glucose importation into skeletal muscle. If this was the case, then this increase in glucose importation initially should induce skeletal muscle NADH production as a result of 2-glyceraldehyde-3-phosphate conversion to 2–1,3 diphosphoglycerate and secondarily through elements of the tri-carboxycyclic (TCA) acid cycle of the mitochondria (MT) in the conversion to pyruvate. TCAP-1-treated muscle significantly (*p* < 0.05) increased static NADH-staining compared to vehicle (Figures 4C,D) supporting a role in increased TCAP-1-mediated-glucose transport and potential MT activity. These static NADH studies were corroborated by subsequent *in vitro* studies (see Section 3.2).

#### 3.1.3 Muscle contractility and fatigue

**FIGURE 5:**
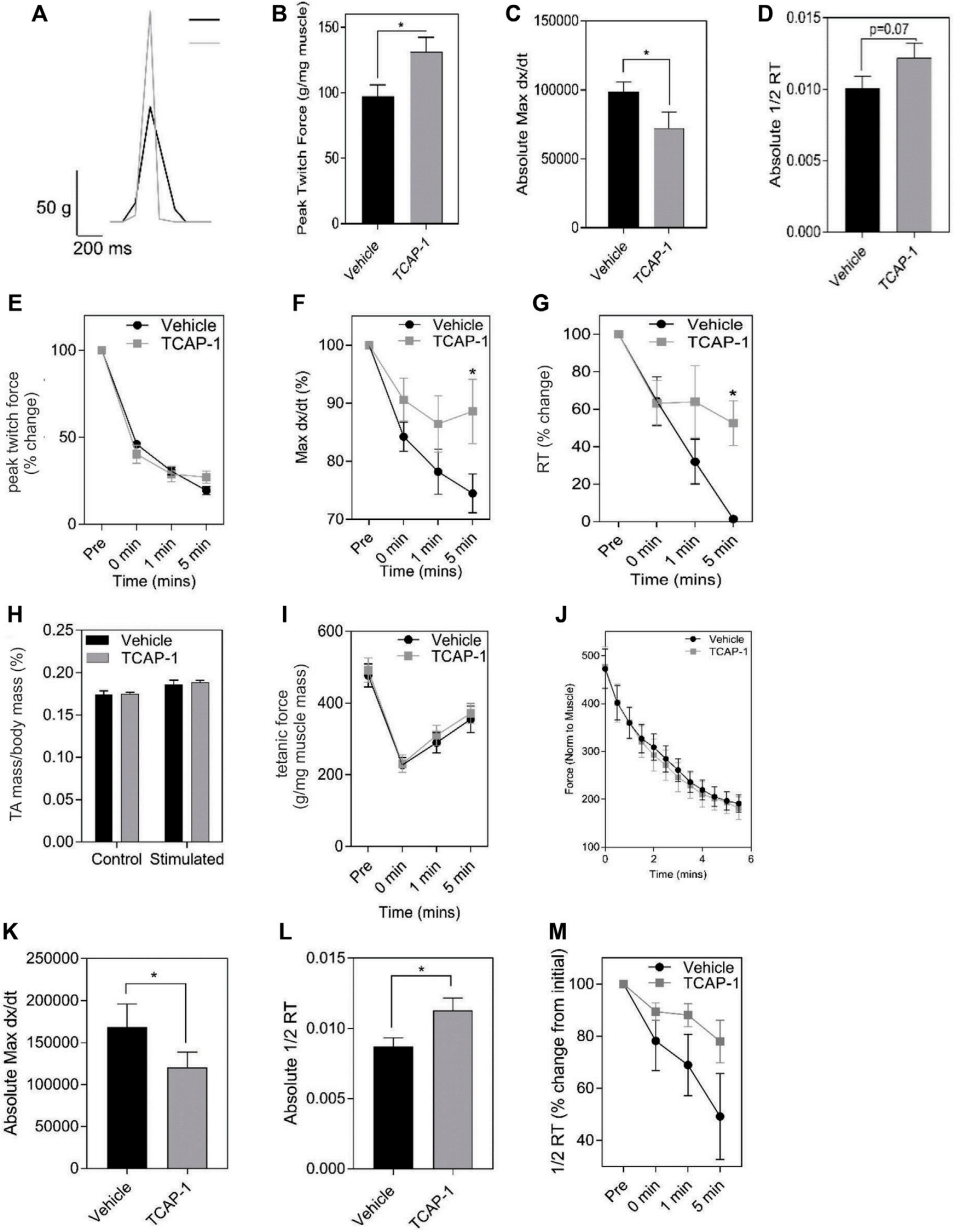
*In vivo* actions of TCAP-1 on rat TA muscle kinetics. **(A–J)**. Muscle twitch kinetics after animals treated with TCAP-1 once a day for 5 days before testing **(A)**. Representative twitch traces (black, vehicle; gray, TCAP-1). **(B)**. Baseline contraction kinetics (*t*-test). **(C)**. Contraction velocity (*t*-test). **(D)**. Relaxation rate (*t*-test). **(E)**. Peak twitch force (2-way ANOVA). **(F)**. Twitch max dx/dt (2-way ANOVA). **(G)**. 1/2RT analysis (2-way ANOVA). TCAP-1 treatment did not affect muscle weight (*t*-test) **(H)**, tetanic force (*t*-test) **(I)** or fatigue force over time **(J)** (*n* = 7–8). **(K**–**M)**. Long term treatment of TCAP-1 on rat hind-limb twitch kinetics. **(K)**. contraction max dx/dt. **(L)**. 1/2RT rate. (**M)**. Relaxation rate.

Muscle performance is related to the amount of energetic substrates available. Therefore, we examined the role of TCAP-1 on muscle activity *in vivo* in rats by determining the efficacy of TCAP-1-mediated contractility using electrical stimulation of the TA muscle. After a 5-day daily treatment of either vehicle or TCAP-1, following a 3-day washout period, muscle contractility was assessed. TCAP-1-treated animals showed improved muscle dynamics where TCAP-1-treated animals exhibited enhanced baseline contraction kinetics with respect to increased peak twitch force (*p* < 0.05) (Figures 5A,B), slower contraction velocity (*p* < 0.05) (Figure 5C), and potentially higher faster relaxation rates (Figure 5D) compared to vehicle-treated animals. Following baseline measurements, a 6-m fatigue protocol was induced to establish the time line of the contractive force in the muscle where contractile kinetics were recorded at 0, 1, and 5 m after the fatigue protocol. TCAP-1 enhanced recovery from the twitch stimulation (Figures 5E–G). Although TCAP-1 did not influence peak twitch force (Figure 5E), it significantly (*p* < 0.05) maintained twitch max dx/dt (Figure 5F) and 1/2RT (Figure 5G) over the course of the fatigue protocol which was diminished in vehicle-treated animals. All data were normalized to muscle mass. The treatment did not affect muscle mass (Figure 5H), tetanic force (Figure 5I) or the fatigue force curve (Figure 5J). Thus, TCAP-1 enhanced the efficiency of the existing muscle morphology, rather than increasing muscle mass, and maintained contraction cycling efficiency during fatigue. To assess the effects of a long-term (LT) treatment, rats were administered either vehicle or TCAP-1, for 3 months (1 injection/week). At 2w post-treatment, the TCAP-1-treated animals elicited a comparable peak twitch force to vehicle-treated animals (Figures 5J,K), however, had significantly (*p* < 0.05) slower contraction velocity and faster (*p* < 0.05) relaxation rate (Figures 5L,M).

### 3.2 *In Vitro* mouse cell studies

#### 3.2.1 Molecular studies

**FIGURE 6:**
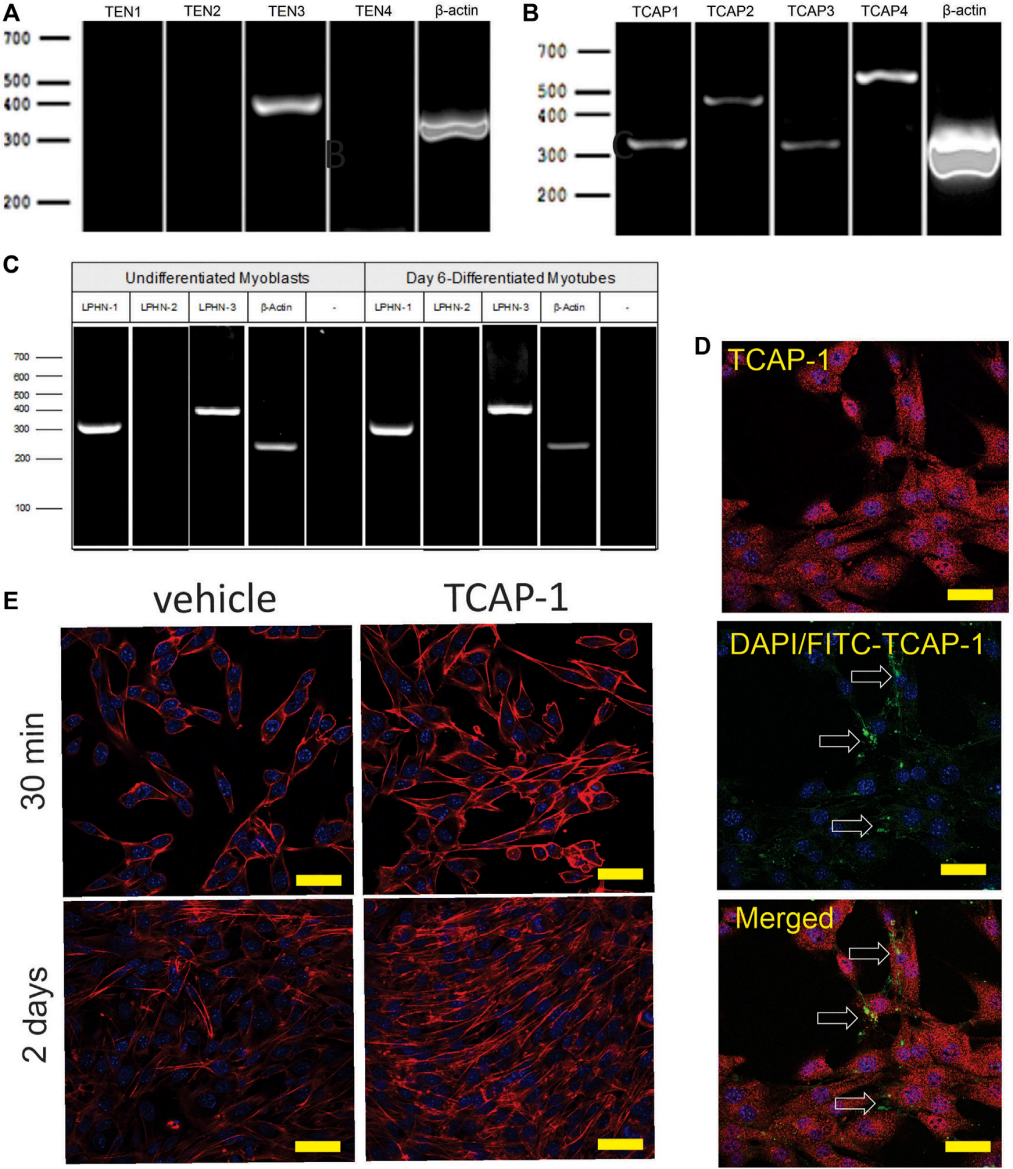
Expression of teneurin, TCAP and LPHN in C2C12 myoblasts. **(A)**. PCR-based teneurin expression; **(B)**. PCR-based TCAP expression; **(C)**. PCR-based LPHN expression. **(D)**. C2C12 cells labelled with TCAP-1 antisera showing the difference between the endogenous ir-TCAP and the presence of FITC-TCAP-1 localization. ir-TCAP-1 is indicated in red, whereas the DNA-associated DAPI labelling is indicated in blue. FITC-labeled TCAP-1 is shown in green. Arrows indicate regions of FITC-TCAP-1 uptake. Scale bars indicate 50 μM **(E)**. Actions of TCAP-1 on the proliferation of the C2C12 myoblasts when treated with TCAP-1 at 30 min and 2 days. Actin is indicated in red, whereas the nuclei are indicated in the DAPI-based blue. Scale bars indicate 100 µM.

The initial PCR screen of C2C12 cells indicated that, although only teneurin-3 was highly expressed (Figure 6A), all 4 TCAP transcripts could be discerned (Figure 6B). In both undifferentiated C2C12 myoblasts, and 6-day myotubules, the transcripts for LPHN-1 and -3 were present (Figure 6C). ICC expression of TCAP-1 showed a similar punctate expression in the cytosol of the ir-TCAP-expressed C2C12 myoblasts (Figure 6D) as we have previously shown for neurons (Chand et al., 2012). In contrast, FITC-labelled TCAP-1 was present at several sarcolemmic regions consistent with the expected expression of the receptor but again similar to what was previously observed in neurons (Chand et al., 2012; Chand et al., 2013b; Husic et al., 2019). Moreover, because TCAP-1 regulates actin organization and polymerization in neurons (Chand et al., 2012), the C2C12 cells were treated with TCAP-1 and examined using the phalloidin stain to highlight actin fibers (Figure 6E). This treatment resulted in a major increase in actin polymerization in the TCAP-1-treated cells at both 30 m (*p* < 0.01) and 2 days (*p* < 0.001).

#### 3.2.2 Caffeine C2C12 viability studies

**FIGURE 7:**
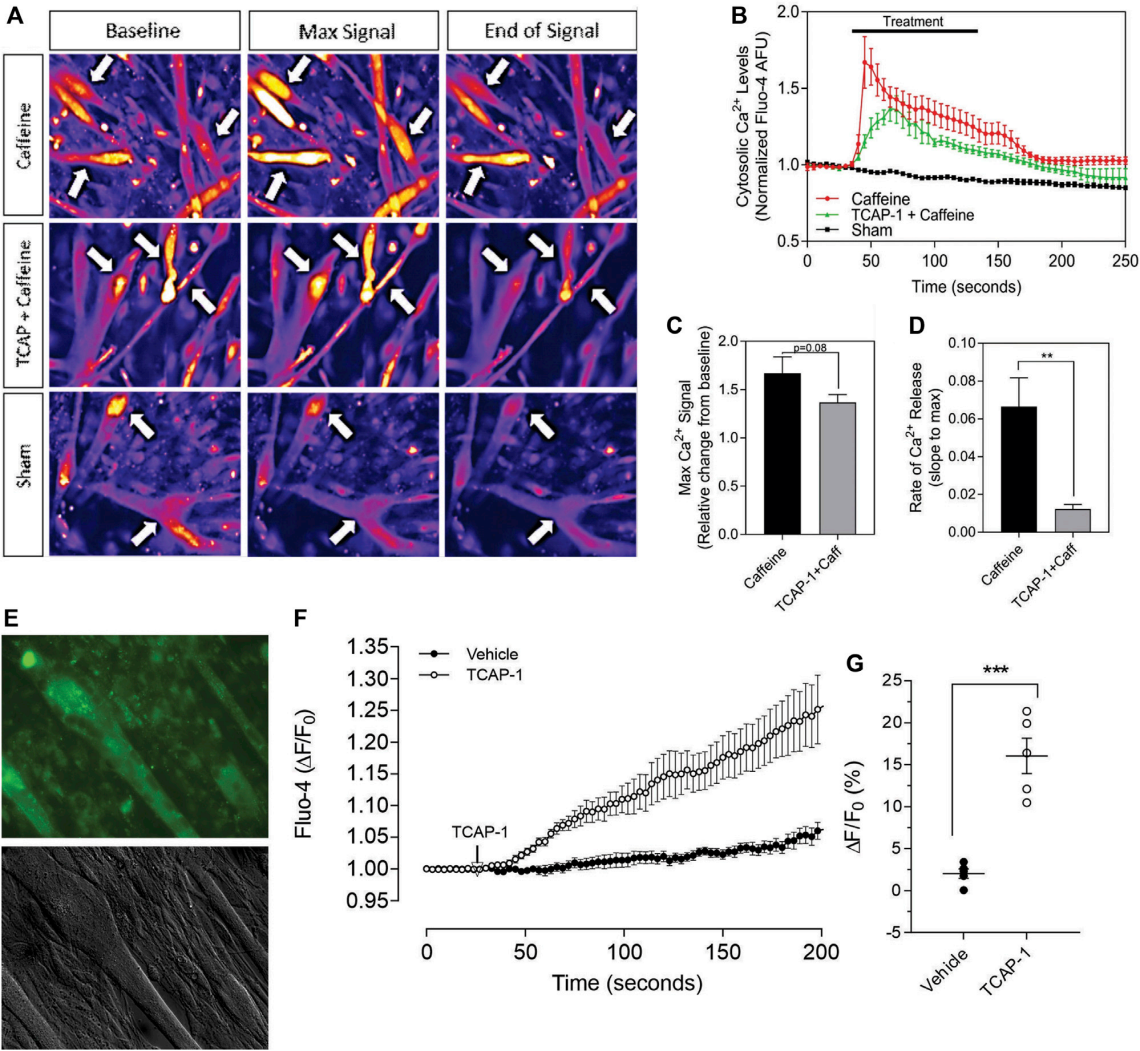
Caffeine- and TCAP-1- mediated Ca^2+^ response in differentiated C2C12 myocytes. **(A)**. Heat-map images showing the Ca^2+^ response induced by caffeine, TCAP-1 or vehicle. Mean and SEM is indicated. **(B)**. Dynamic concentration changes over the period of analysis shown in “A”. Mean ± SEM is indicated. **(C)**. Total concentration changes of the manipulations of the study period indicated in “A”. **(D)**. Rate of Ca^2+^ release between caffeine and TCAP-1. **(E)**. TCAP-1 mediated Ca^2+^ actions show normal morphology in cells. **(F)**. Rate of increase in Ca^2+^-associated fluorescence after administration of TCAP-1. **(G)**. Quantification of the change in TCAP-1 mediated intracellular Ca^2+^ concentrations indicated in “F”. Significance was determined by a Students t-test. (* *p* < 0.05; **p < 0.01; ***p < 0.001; ****p < 0.0001. Mean ± SEM indicated *n* = 4). Note that in this Figure, that F/F0 has been set to “1” for clarity.

Having established that TCAP-1 behaved in a similar manner as previously shown in neurons, the viability of the Ca^2+^ response in the differentiated myotubules was evaluated to determine their efficacy before proceeding to further studies. Initially, caffeine was used to determine the potential limits of the Ca^2+^ response in the differentiated myotubules relative to the TCAP-1 response (Figures 7A–D). These studies indicated that the myotubules were active and viable, and with respect to the Ca^2+^ response, did not show an appreciable decrease in cytosolic Ca^2+^ concentrations (Figure 7B), although it did attenuate the rate of cytosolic Ca^2+^ concentrations (*p* < 0.01). Taken together, these studies indicated that the myotubules were viable with respect to our preparation, and that the attenuating TCAP-1 response indicated that additional regulating factors were likely present. Thus, given these observations, the direct action of TCAP-1 on Ca^2+^ flux in myotubules was examined (Figures 7E–H). TCAP-1 increased Ca^2+^ concentrations by almost 4-fold relative to the control-treated cells (*p* < 0.001).

#### 3.2.3 Glucose transporter studies

**FIGURE 8:**
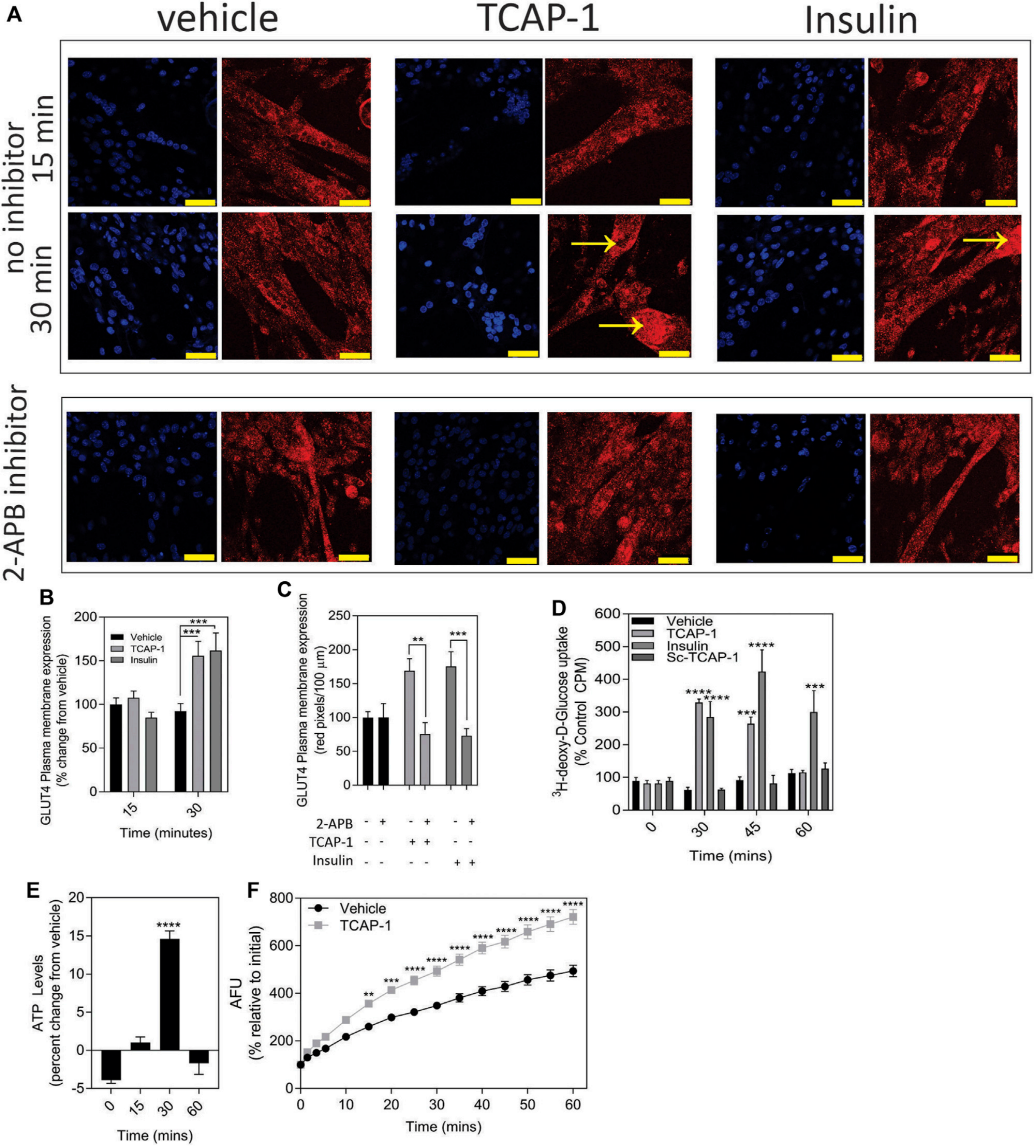
TCAP-mediated glucose metabolism in C2C12 cells. **(A)**. Regulation of ir-GLUT4 by TCAP-1 and insulin myotubules. Red indicates ir-GLUT4 whereas blue shows DAPI staining of the nuclei. Arrows indicated regions of high immunoreactivity. Scale bar = 100 µM. **(B)**. Quantification of ir-GLUT4 expression over 30 min. **(C)**. Effect of the IP3R inhibitor, 2-APB on TCAP-1- and insulin-mediated ir-GLUT4 labelling. **(D)**. Uptake of ^3^H-2-deoxyglucose in C2C12 myoblasts by TCAP-1 and insulin. **(E)**. Changes in static ATP concentrations following treatment by TCAP-1. **(F)**. NADH production increase as determined by a resourin assay following TCAP-1 treatment relative to the vehicle. Significance was determined by a *t*-test as indicated in **(C, F)**, or one-way ANOVA shown in B D and E. (* *p* < 0.05; **p < 0.01; ***p < 0.001; ****p < 0.0001. Mean ± SEM indicated *n* = 6).

In skeletal muscle, the predominant glucose transporter protein (GLUT) isoform is the insulin-sensitive GLUT4 protein. Using insulin as a control, a significant (*p* < 0.001) increase in the expression of the ir-GLUT4 transporters was observed for both TCAP-1 and insulin treatments over 30 m (Figures 8A,B) in C2C12 cells. To determine whether this ir-GLUT4 response was dependent on the TCAP-1-mediated Ca^2+^ release, the IP3R antagonist, 2-APB, that abolishes the TCAP-1 Ca^2+^ response, was investigated. In the presence of the inhibitor, both TCAP-1 (*p* < 0.01) and insulin (*p* < 0.001) inhibited the ir-GLUT4 expression (Figures 8A,C). This importation of glucose by TCAP-1 was further corroborated in C2C12 cells showing that TCAP-1 significantly (*p* < 0.0001) induced ^3^H-2-deoxyglucose increase into the cytosol over 30m, similar to that of insulin (Figure 8D). However, both peptides show distinct glucose-uptake profiles; whereas insulin induced a significant increase at 30 m (*p* < 0.001), 45 m (*p* < 0.001) and 60 m (*p* < 0.01), TCAP-1 increased glucose uptake at 30 m (*p* < 0.001) but was attenuated by 45 m (*p* < 0.01) and returns to baseline at 60 m. The scTCAP-1 treatment, used separately as a negative peptide control in this study, showed no significant change from the saline vehicle.

#### 3.2.4 ATP and NADH turnover studies

Increased glucose importation increases ATP and NADH turnover in cells due to glycolytic and tricylic acid (TCA) cycle activity. Therefore, this was examined with respect to TCAP-1 treatment. As a result, both ATP (*p* < 0.001) (Figure 8E) and NADH (*p* < 0.001) (Figure 8F) turnover were significantly increased after 30 m of TCAP-1 treatment, although NADH levels remained about 60% higher (*p* < 0.001) than vehicle levels after 60 m.

**FIGURE 9:**
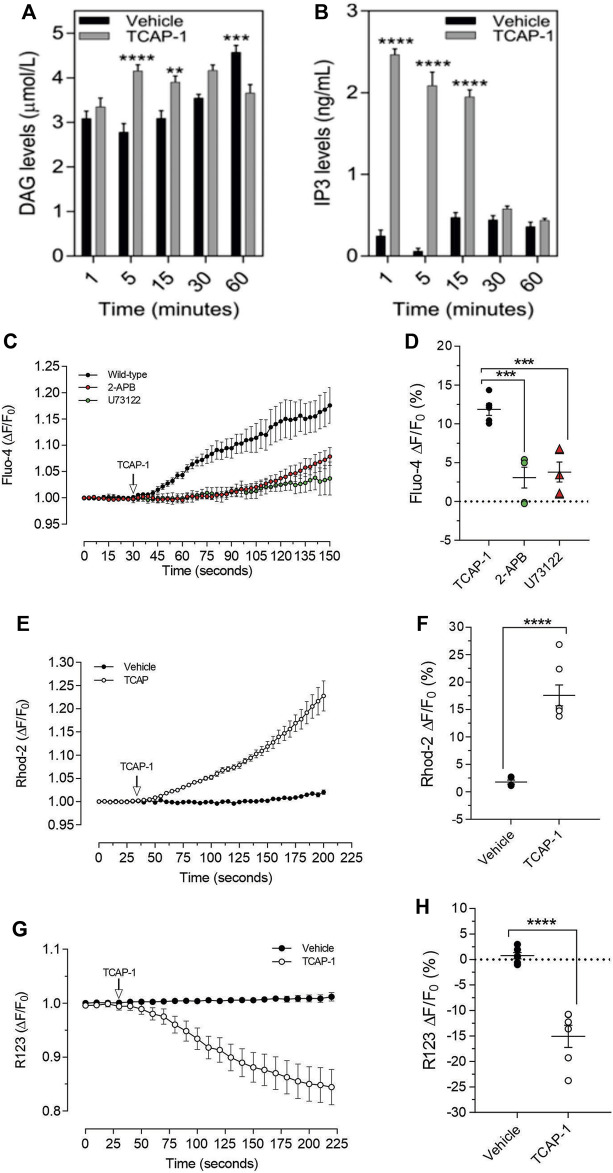
**T**CAP-1 mediated calcium regulation in C2C12 cells. **(A)**. TCAP-1 mediated increase in intracellular DAG concentrations (mean and SEM shown; *n* = 6). **(B).** TCAP-1 mediated increase in intracellular IP3 concentrations (mean and SEM shown; *n* = 6). **(C).** Increase in intracellular TCAP-1 mediated Ca^2+^ concentrations and inhibition by the IP3 receptor (2-APB) and phospholipase C (U73122) antagonists; **(D)**. Quantification of data shown in C (*n* = 6). **(E).** Uptake in Ca^2+^-mediated Rhod-2 into mitochondrial membranes. **(F)**. Quantification of data shown in “E” based on the change at 200 s. **(G)**. Decrease in Rhod-123 immunofluorescence in mitochondrial membranes as a result of TCAP-1 administration. This decrease in Rhod-123 indicates a decrease in mitochondrial membrane depolarization. **(H)**. Quantification of the data indicated in G based on the changes at 225 s (* *p* < 0.05; **p < 0.01; ***p < 0.001; ****p < 0.0001.) Note that in this Figure, that F/F0 has been set to “1” for clarity.

As we have previously established that the IP3-DAG pathway is important for TCAP-1-mediated intracellular Ca^2+^ flux in neurons, this pathway was examined in C2C12 cells. Relative to the vehicle, TCAP-1 induced a significant increase at 5 and 15 m (*p* < 0.001; Figure 9A) in intracellular DAG concentrations and a major increase between 1 and 15 m (*p* < 0.0001; Figure 9B) in IP3 concentrations. To confirm this action, TCAP-1-treated C2C12 cells were blocked with either the IP3R antagonist, 2-APB, or the phospholase C inhibitor, U73122. The 2-APB and U73122 treatment reduced TCAP-1-mediated Fluo-4 concentrations to about 30% (*p* < 0.01) of their original values indicating that the IP3-DAG pathway plays an active role in increase of TCAP-1-mediated intracellular Ca^2+^ flux (Figures 8C,D). Because this TCAP-1-mediated rise in intracellular Ca^2+^ concentrations can target the MT (Hogg et al., 2018), the MT Ca^2+^ dye, Rhod-2 was utilized to determine the concentration of Ca^2+^ sequestration in MT. There was a 5-fold increase (*p* < 0.001) in Rhod-2-associated Ca^2+^ labelling over 200s (Figures 9E,F). Related to this, Rhod-123, was used to determine the level of MT polarization. TCAP-1 treatment significantly decreased Rhod-123 fluorescence (*p* < 0.001) relative to vehicle indicating depolarization of the MT membrane (Figures 9G,H).

#### 3.2.5. Attenuation of LPHN by siRNA and CRISPR methods

**FIGURE 10:**
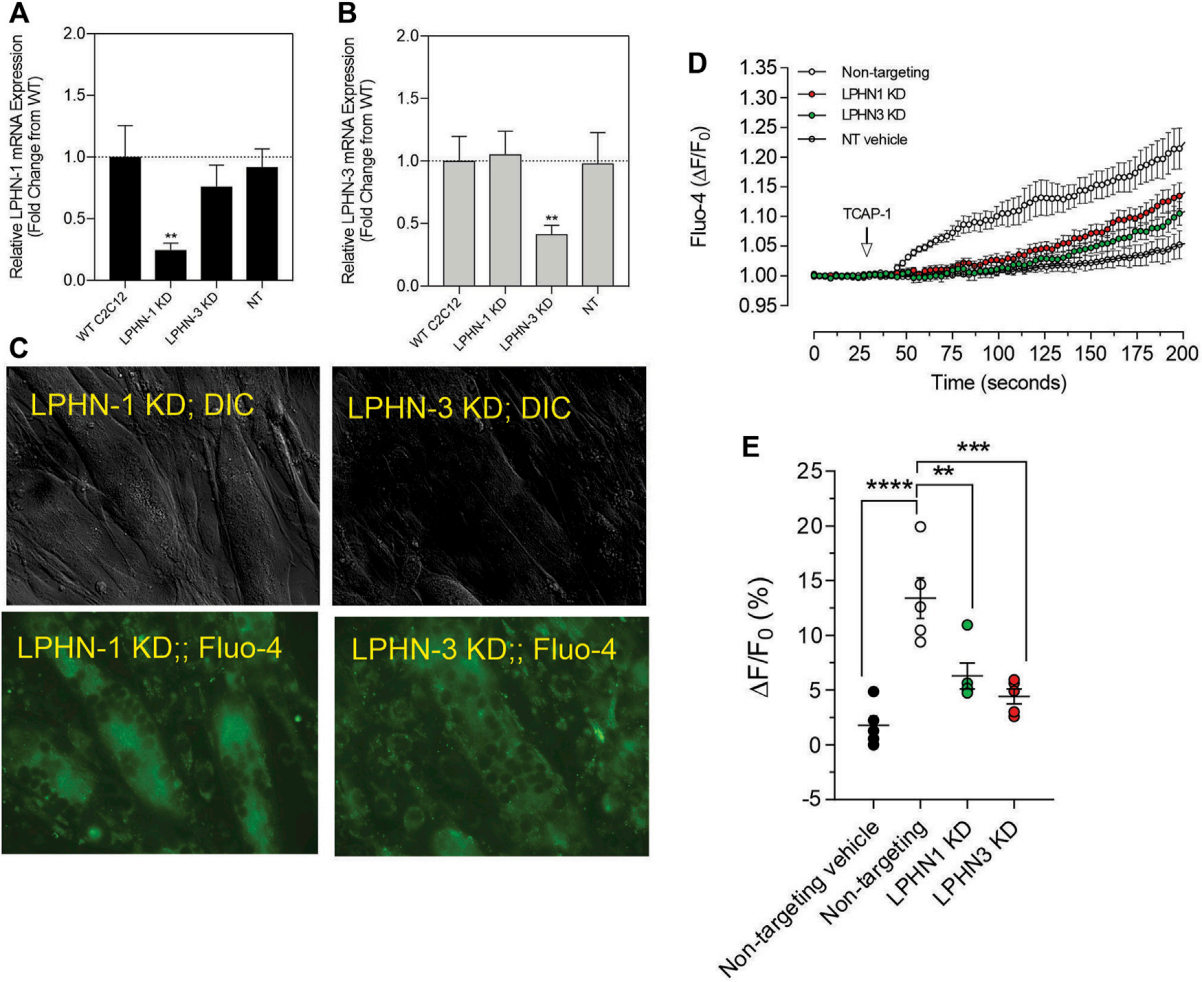
siRNA knockdown of LPHN-1 and -3 in C2C12 cells. **(A)**. Cells transfected with the LPHN-1-targetting siRNAs showed a significant reduction (*p* < 0.01) in LPHN-1 mRNA expression. **(B)**. Cells transfected with the LPHN-3 targeting mRNA significantly (*p* < 0.01) reduced LPHN-3 mRNA expression. **(C)**. Cells treated with either siRNAs showed normal morphology. **(D)**. Changes in Ca^2+^ accumulation in cells transfected with either LPHN-1 or -3 siRNA oligonucleotides. **(E)**. Quantification of the data shown in “D” at 200 s.

**FIGURE 11:**
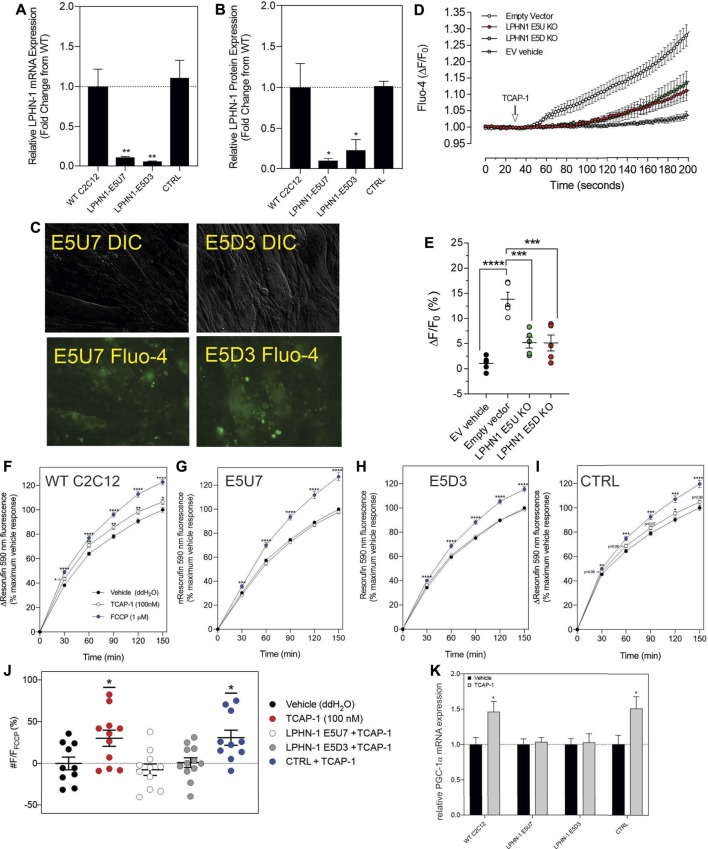
CRISPR-based knockouts of the LPHN-1 and -3 genes in C2C12 cells. The two clones (E5U7) **(A)** and (E5D3) **(B)** significantly reduced LPHN-1 expression relative to NT and WT cells. **(C)**. The morphology of the C2C12 cells were normal in both transfected cell lines. **(D)**. Changes in cytosolic Ca^2+^ accumulation in the various cell types. **(E)**. Quantification of the data shown in “D” after 200 s. **(F–I)**. Changes in NADH production as determined by the resasurin assay where the WT cells show a significant increase in the NADH-mediated resorufin fluorescence but is ablated in the E5U7 **(G)** and E5D3 **(H)** KO clones, but is retained in the null vector (NT) clone **(I)**. **(J)**. Quantification of the data shown in **(F–I)**, **(K)**. Reduction in PGA-1α mRNA expression by PCR in E5U7 and E5D3 clones. * *p* < 0.05; **p < 0.01; ***p < 0.001; ****p < 0.0001. Mean ± SEM indicated *n* = 7–8. Note that CTRL refers to the null-vector control and that F/F0 has been set to “1” for clarity.

Although these studies are similar with previous investigations of teneurins, TCAPs and LPNHs in neurons, this is the first study to examine teneurin/TCAP and LPHN activity in skeletal muscle function. To determine whether the TCAP-1 activation was dependent upon the LPHN receptors, these genes were knocked-down (KD) using siRNA oligonucleotides, or knocked-out (KO) using CRISPR in the C2C12 cells. Despite the modification of receptor expression in these methods, cell morphology was essentially identical to the morphology of the cells indicated in Figure 7. Using the C2C12 cells, the LPHN-1 and -3 expression was reduced using the siRNA oligonucleotides. The LPHN-1 receptor mRNA was reduced about an 80% (*p* < 0.01) relative to the WT cells. Transfection with either the LPHN-1 siRNAs or the null-vector (NT) did not significantly change mRNA expression relative to the WT control (Figure 10A). Similarly, the LPHN-3 siRNA-associated oligonucleotides significantly (*p* < 0.01) decreased its mRNA expression about 65% relative to the WT cells. There were no significant changes in mRNA expression of the LPHN-1 transcript in either the LPHN-1 KD or the NT cells (Figure 10B). Despite the reduced expression of these receptors, cell morphology was normal (Figure 10C). TCAP-1 increased cytosolic Ca^2+^ in cells transfected with the NT control, however, relative to the NT control, TCAP-1 did not increase Ca^2+^ in either the LPHN-1 and -3 siRNA-transfected cells, which showed a significant decrease (*p* < 0.01 and *p* < 0.001, respectively) in intracellular Ca^2+^ concentrations (Figures 10D,E). However, because both LPHN-1 and -3 siRNA oligonucleotides unexpectedly reduced intracellular Ca^2+^ concentrations by similar amounts, we repeated this study by ablating the LPHN-1 and -3 receptors using CRISPR methods. The E5U7 target reduced LPHN-1 expression by about 90% (*p* < 0.01) whereas, the E5D3 target reduced mRNA levels by about 95% (*p* < 0.05) relative to the WT cells (Figure 11A). A significant decrease (*p* < 0.05) in LPHN-1 mRNA levels were present in both sets of transgenic cells (Figure 11B). Importantly, cell morphology was normal in the transgenic cells (Figure 11C). CRISPR-based KOs of the LPHN-3 gene were unsuccessful after numerous attempts hence studies were performed with the LPHN-1 KOs only. Similar to that achieved using the siRNA knock-down cells, both CRISPR-associated KO transgenic cells (E5U and E5D) reduced intracellular Ca^2+^ levels by about 60% (*p* < 0.001) (Figures 11D,E). Taken together, both the siRNA- and CRISPR-associated methods to reduce mRNA expression indicate that the TCAP-1 associated intracellular Ca^2+^ flux was mediated primarily by its interactions on LPHN-1 and -3.

To corroborate these findings with the previous *in vivo* observations that TCAP-1 could regulate NADH turnover in skeletal muscle cells, the action of TCAP-1 on NADH production *via* a resazurin assay was performed on the CRISPR LPHN KO cells (Figures 11F–I). From 30 to 150 m, the TCAP-1-treated WT C2C12 cells showed a significant increase (*p* < 0.01; *p* < 0.05, respectively) in fluorescence compared to the vehicle (Figure 11F) indicating an increase in NADH metabolism. In contrast, with respect to the KO cells, LPHN-1 E5U7 (Figure 11G) and E5D3 (Figure 11H) the TCAP-1-mediated increase of NADH production was ablated, whereas the NT control cells showed a significant (*p* < 0.01) increase after 120 m (Figure 11I). FCCP treatment, indicating cell viability induced significant increases (*p* < 0.001; *p* < 0.0001) across all cell types (Figures 11F–I) where the relationship of the TCAP-1 treated cells to that of the FCCP-treated cells was consistent. These data indicate that reduction of LPHNs result in the ability of TCAP-1 to stimulate NADH activity.

Given that the *in vivo* studies showed that TCAP-1 can modulate MHCI and that TCAP-1 may also affect other hormones and signalling factors, it was unclear if the purported fiber changes observed in TA muscle were a direct result of TCAP-1. Therefore, because PGC-1α is a transcription factor that up-regulates MHCI expression (Liang and Ward, 2006), the TCAP-1 actions on PGC-1α were measured by qRT-PCR. Following the TCAP-1 treatment (100 nM), neither the E5U7 nor the E5D3 cells showed any significant increases, whereas, both the WT cells and NT controls showed about a 50% increase (*p* < 0.05) in PGC-1α expression (Figure 11K). This indicated that TCAP-1 has the potential to directly influence MCH fiber expression at the transcriptional level.

## 4 Discussion

This study describes a novel mechanism underlying skeletal muscle physiology and is the first to show a functional relationship between teneurins and latrophilins (LPHN) with respect to skeletal muscle function in mammals using rodent models. We have previously established that there is a functional peptide on the distal tip of the teneurin extracellular region (teneurin C-terminal associated peptide; (TCAP) and is highly active in the CNS. Now, our data indicates that TCAP-1 affects skeletal muscle strength and fatigue, *in vivo, via* a glucose-associated and, likely, an aerobic MT-based mechanism. Moreover, TCAP-1 interacts with the putative teneurin receptors, LPHN-1 and -3 to activate the PLC-IP3-DAG pathway to regulate intracellular Ca^2+^ flux that ultimately regulates glucose importation and MT activity similar to what we have previously shown in the CNS. The results from our *in vivo* studies were subsequently tested *in vitro* utilizing the rat skeletal cell line, C2C12, to establish a cellular model upon which to base the *in vivo* actions of TCAP-1 with respect to skeletal muscle dynamics.

A critical aspect of this study was the utilization of TCAP-1 as a peptide analog of the distal C-terminal region of teneurins. The genomic structure of the TCAP region of teneurins indicated that it possessed a potentially cleavable peptide (Wang et al., 2005; Jackson et al., 2018). A synthetic version of TCAP-1 was developed by replacing the N-terminal glutamine with pyroglutamyl acid and amidating the C-terminal residue (Wang et al., 2005). The resultant synthetic peptide was highly efficacious at regulating neural function, behavior and reproductive physiology in rodents (Wang et al., 2005; Al Chawaf et al., 2007a; Silva et al., 2011; Tan et al., 2011; Chand et al., 2012; Chand et al., 2013b) indicating that TCAP-1, itself, possessed independent biological functions. Thus, rat/mouse TCAP-1 was utilized in this study based on our previous work with this peptide. As indicated in Figure 1, the primary structure of rat and mouse TCAP-1 is identical, and consequently, the same peptide was utilized for all *in vivo* and *in vitro* experiments. However, it is important to point out that, although there are 4 paralogous forms of teneurins and TCAP in vertebrates, albiet, with significant primary structure conservation among them, our study has utilized only TCAP-1. TCAP-1 was utilized, therefore, as a proxy for all TCAPs present in the organism or tissue, to determine the potential to regulate skeletal muscle function. Based on our previous studies of this peptide and its level of primary structure conservation, evidence indicates that our supposition is valid.

Elucidation of the teneurin/TCAP-LPHN network is complex. To date, there are 4 teneurins found in vertebrates, each of which possesses a TCAP at its extracellular tip (Wang et al., 2005; Lovejoy et al., 2006; Tucker and Chiquet-Ehrismann, 2006; Young and Leamey, 2009; Chand et al., 2013a; Michalec et al., 2020). Although the three latrophilin paralogs (LPHN-1 to 3) have been identified in the vertebrate genome (Davletov et al., 1996; Fredricksson et al., 2003; Silva and Ushkaryov, 2010) the stoichiometry among teneurins and the LPHNs has been only partially resolved. Both ligand and receptor proteins possess multiple domains that interact with a variety of peripheral ligands in the extracellular matrix, the membrane and cytosol (Baumgartner et al., 1994; Levine et al., 1994; Oohashi et al., 1999; Jackson et al., 2018; Li et al., 2018). TCAP, as an amphiphilic peptide that may be cleaved (Jackson et al., 2018; Li et al., 2018), or expressed separately (Chand et al., 2013b; Reid et al., 2021) is a “wild-card” in this perplexity of molecular interactions. Although TCAP-1 is clearly bioactive with respect to cytoskeletal reorganization (Al Chawaf et al., 2007a; Chand et al., 2012; Chand et al., 2013b; Husic et al., 2019), glucose regulation (Hogg et al., 2018; Lovejoy and Hogg, 2020), signal transduction (Qian et al., 2004; Wang et al., 2005; Chand et al., 2012; Hogg et al., 2018), metabolism (Wang et al., 2005; Hogg et al., 2018; Husic et al., 2019; Reid et al., 2021) and stress-associated behavior (Al Chawaf et al., 2007b; Rotzinger et al., 2010; Kupferschmidt et al., 2011; Tan et al., 2011; Tan et al., 2012; Erb et al., 20142014), these studies have focused at the neurological level. Despite this emphasis, few studies (Ishii et al., 2015) regarding the role of teneurins/TCAP and LPHN on skeletal muscle physiology have been reported.

Our goal in examining the role of TCAP-1 and LPHN-1 and -3 was not intended to establish a specific molecular interaction, *per se*, but rather to show that the teneurin/TCAP-LPHN system plays an important role in skeletal muscle metabolism. Consequently, our investigation that paralogs of teneurins, TCAPs and LPHNs were present both in hind-limb skeletal muscle preparations and in C2C12 cells provided the basis of this molecular system with respect to skeletal muscle physiology. Because TCAP-1 co-localization to cell membranes was associated with the dystroglycan (DG) complex (Chand et al., 2012), we used DG antibodies to delineate the sarcolemma. In this study, the interaction of the teneurin-3 and LPHN-1 in the TA muscle occurred at specific nodes in the sarcolemma rather than being spread throughout all regions of DG labelling (see Figure 3B). Our data indicates that teneurin-3 is the dominant teneurin in skeletal muscle tissue. However, the antisera available for the teneurins limited us to visualize histochemically only teneurin-3-like epitopes, although PCR expression indicated that the teneurin-3 transcript was expressed. All 4 TCAPs were expressed by PCR although TCAP-3 showed low expression. In contrast, expression of these transcripts in C2C12 cells indicated high expression of teneurin-3 along with all 4 TCAPs (see Figures 6A,B). This is the first time we have observed high TCAP expression without the corresponding teneurin expression using PCR although we have only focused on neurons previously. We acknowledge that it can be difficult to reconcile the expression patterns of teneurins using the antibodies available at the time, however, it is important that both immunoreactive epitopes are present. These data may indicate a fundamental difference among teneurin and TCAP expression in skeletal cells relative to neurons. For example, TCAP-1 immunoreactivity in the C2C12 cell cytosol is consistent with the cytosolic distribution of TCAP-1 immunoreactivity in immortalized neurons (Chand et al., 2012), FITC-TCAP-1 interaction sites show in C2C12 cells show only punctate regions of aggregation on or near the sarcolemma, whereas in immortalized neurons, extended regions along the plasma membrane show FITC-TCAP-1 congregation. This may reflect the polar organization of neurons relative to skeletal muscle cells.

Our skeletal muscle data indicates that TCAP-1, like in neurons (Hogg et al., 2018), regulates glucose-mediated aerobic-based energy metabolism. Several observations support this hypothesis. First, *in vivo*, ir-LPHN primarily labeled small and intermediate muscle fibers in the rat tibialis anterior (TA) muscle (see Figures 3C,D), that are typically associated with aerobic action. Second, with respect to MHC proteins, TCAP-1 induced the greatest increase in MCHI expression in both short-term and long-term TCAP-1 treatment of rats. Muscle kinetics are dependent, in part, on the relative proportion of muscle fiber types. Although the TA muscle consists of 95% type-II muscle (fast-twitch glycolytic muscle fiber type), TCAP-1 imparts traits of type-I fibers (slow-twitch and oxidative) which possess greater MHC expression. Importantly, both short-term (*p* < 0.01) and long-term TCAP-1 (*p* < 0.01) administration (Figures 3E,F) increased in the MCHI fiber expression, although in short-term TCAP-1- treated animals, MHCII expression was specifically decreased in MHCIIa (*p* < 0.05); MHCIIx (*p* < 0.01) and MHCIIb (*p* < 0.05) mRNA expression, where only MHCIIB showed a decrease (*p* < 0.01) in the long-term treated rats. These expressional changes of MHC transcription were corroborated by the expression of PGC-1α, a critical transcriptional co-factor that regulates the MT actions of myosin chain transcription. Using the C2C12 cells as a model, TCAP-1 increased the transcription of PGC-1α and was inhibited (*p* < 0.05) by CRISPR-mediated KOs of the LPHN-1 gene. Thus, the TCAP-1-mediated Ca^2+^ surge may activate CaMKIV and CaN transcriptional regulators to promote the transcription of PGC-1α. Overall, this indicates that TCAP-1 is increasing slow-twitch gene expression, consistent with the *in vivo* contractile kinetics observed in Figure 5. Third, rats treated with TCAP-1 enhanced baseline contractile kinetics under basal and fatigue conditions using both short- and long-term TCAP-1 administration. Fourth, a single dose of TCAP-1 increased uptake of ^18^F-2-deoxyglucose in rat hind-limb regions as determined by fPET analysis. Fifth, because Ca^2+^ and ATP are required for proper muscle contraction initiation and relaxation, TCAP-1 significantly increased ir-GLUT4 expression (Figure 8A), increased ^3^H-2-deoxyglucose uptake (Figure 8D), ATP (Figure 8E) and NADH (Figure 8F) using the C2C12 myoblasts and myotubules. Taken together, these studies support the hypothesis that TCAP-1 regulates glucose uptake and metabolism in skeletal muscle cells in a similar manner previously described in neurons (Hogg et al., 2018). However, the possibility that TCAP-1 regulates free fatty acid (FFA) metabolism in skeletal muscle given its action on the MT cannot be discarded. Although this was beyond the scope of the current investigation, this possibility should be investigated in the future.

Glucose is the main energy nutrient for skeletal muscle function, thus, up-regulation of glucose metabolism could impact skeletal muscle activity. Therefore, the influence of TCAP-1 on these contractile kinetic parameters indicates that TCAP-1 may modulate Ca^2+^ levels to enhance sarcoplasmic reticulum (SR)-sarcomere coupling. During fatigue, cytosolic Ca^2+^ levels accumulate due to inefficient SR-sarcomere coupling, thereby reducing muscle function as was observed in contractile kinetic parameters such as peak twitch force and 1/2RT. TCAP-1 treatment significantly increased both parameters, indicating a clear role in Ca^2+^ modulation. Moreover, these results also suggest that TCAP-1 increases ATP production rate to meet the energetic demands of the muscle during fatigue. After the contraction, Ca^2+^ is cleared from the cytosol and re-uptaken into the SR *via* the associated Ca^2+^ -ATPase (SERCA) pumps. SERCA pumps are high energy-consuming channels that account for 20–50% of the energy turnover in a single contraction cycle (Calderón et al., 2014; Holwerda and Locke, 2014). Prolonged stimulation results in the rapid depletion of ATP, thus reducing SERCA activity, ultimately leading to the accumulation of cytosolic Ca^2+^. However, because TCAP-1 increases glucose uptake into the muscle, it provides additional substrates for energy metabolism which could maintain SERCA activity during fatigue. This is corroborated by the finding that TCAP-1 had significantly faster 1/2RT during fatigue compared to vehicle treatment and by the increase in the type-1 muscle fiber-associated gene transcription that occurred in both short-term and long-term actions of TCAP-1.

Although we have established an indirect action of TCAP-1 on MT activity the current study does provide evidence that TCAP-1 targets the MT as the peptide has potent actions upon Ca^2+^ modulation, NADH production and glucose signaling. As TCAP-1 increases Ca^2+^ uptake into the MT, likely from shuttling Ca^2+^ from the SR, this may stimulate enzymes in the TCA cycle, such glycerol phosphate dehydrogenase, pyruvate dehydrogenase phosphatase, isocitrate dehydrogenase and oxoglutarate dehydrogenase (Wan et al., 1989; Denton, 2009). TCAP-1 may activate MT respiration *via* the ETC and lead to increased energetic output, as observed by increases in ATP and NADH static staining *in vivo* and production *in vitro*. Thus, this may explain, in part, the enhanced cellular metabolism and function results under TCAP-1 treatment. Previous studies in neurons have established that the IP3-DAG pathway is activated in response to TCAP-1. In this study, we showed that a similar situation occurs in C2C12 cells and, likely, in skeletal muscle. TCAP-1 treatment of C2C12 cells increase intracellular Ca^2+^ flux that can be blocked using IP3 receptor (2-APB) and phospholipase C (U73122) inhibitors. Moreover, this increase in intracellular Ca^2+^ is likely responsible for the depolarization of the MT membranes. This work supports a previous report that SR-associated Ca^2+^ release was directed toward the MT in skeletal muscle (Dias-Vegas et al., 2018).

A compelling question arises in this study as to why TCAP-1 has long-term kinetic actions *in vivo* in the order of days, whereas *in vitro* studies indicate the Ca^2+^ response occurs within minutes or less. These new studies in skeletal muscle corroborate with previous studies in neurons and in the brain. We previously showed that TCAP-mediated Ca^2+^ flux occurs within seconds and minutes after administration to immortalized neurons (Hogg et al., 2018) and the cytoskeletal rearrangement occurs within hours (Al Chawaf et al., 2007a; Chand et al., 2012; Chand et al., 2013b), yet *in vivo*, we observed significant increases in hippocampal spine density (Tan et al., 2011), glucose uptake into the brain and corresponding changes in plasma glucose and insulin (Hogg et al., 2018) and behavioral changes (Wang et al., 2005; Tan et al., 2008; Rotzinger et al., 2010; Kupferschmidt et al., 2011; Tan et al., 2011; Tan et al., 2012; Erb et al., 20142014) over a period of days and weeks. In this current study on skeletal muscle, we show a similar situation where the TCAP-1-mediated actions on the signal transduction mechanism and their portential actions on the sarcomere occur within minutes of administration, yet we can measure significant changes in skeletal muscle contractility *in vivo* several days after administration. Now that we have two distinct models that both show a similar temporal response between *in vitro* and *in vivo* studies, we offer a hypothesis. We posit that synthetic TCAP elicits a cascade of events that begin with the activation of the signal transduction pathways associated with its interaction with the latrophilins. Stimulation of the intracellular Ca^2+^ response, in part, along with other potential intercellular messenger systems (i.e. cAMP-PKA) lead to sarcomere function, glucose importation and MT activation, thus extending the transient TCAP-1 action in the cells to a longer functional period. *In vivo*, TCAP may also act to stimulate the expression of teneurins (Wang et al., 2005) to provide a longer term source of TCAP. To be sure, this hypothesis remains mostly speculative based on the data available and numerous questions remain. Importantly, it offers a testable hypothesis on this novel mechanism.

These studies are consistent with previous observations in immortalized mouse neurons (Hogg et al., 2018) and in zebrafish (Reid et al., 2021). Although these previous studies indicate a relationship with TCAP-1 and LPHN action on Ca^2+^ flux involving the IP3-DAG pathway (Rahman et al., 1999; Hogg et al., 2018) other studies indicate that the LPHN-mediated AMP-PKA pathway may also be activated by teneurins and TCAP (Lang et al., 1998; Sando, Südhof). For example, TCAP-1 and -3 can increase cAMP levels in immortalized mouse neurons (Qian et al., 2004; Wang et al., 2005; Al Chawaf et al., 2007a). Further, studies of vertebrate teneurins have also implicated activation of the PKA-cAMP cascade (Silva et al., 2011). However, our goal in this study was to establish a mechanism by which TCAP-1 can regulate energy metabolism in C2C12 cells, and for this reason we have focused on a Ca^2+^-associated mechanism, as it aligns with previous studies. However, we acknowledge that given the complexity of teneurin-LPHN actions, other signal transduction systems such as ERK-MEK (Chand et al., 2012) are also likely required for the full set of teneurin- and TCAP-mediated LPHN actions on cells. Ancient-evolving peptide-protein systems will likely impinge on more than one intracellular signal cascade events because they evolved before many of the later intracellular signaling transducing pathways (Silva et al., 2011).

The MT are ultimately responsible for supplying aerobic-based energy requirements to eukaryotic cells. We have previously shown the relationship of TCAP-1 mediated energy production and the MT in the mouse neurons (Hogg et al., 2018) and in zebrafish metabolism (Reid et al., 2021), however this was the first study to establish the link between the teneurin/TCAP-LPHN system and MT in skeletal muscle cells. Although we have not studied MT respiration directly in this study, previously we showed that TCAP-3-treated zebrafish (Reid et al., 2021) increased both basal and respiratory reserve capacity. Total MT respiration is linked to both proton leak and ATP-linked respiration (Rolfe and Brand, 1997), however no TCAP-3-associated actions on the latter could be detected, although proton leak was increased in these studies. Thus taken together, although these studies support a TCAP-mediated MT action, its effects may vary with the stress and metabolic environment of the organism and species.

The role of the teneurins, TCAP and LPHNs, together, has previously not been examined in skeletal muscle. Therefore, we utilized both siRNA- and CRISPR-based methods to determine if the reduced activity of the LPHN-1 and -3 receptors would attenuate TCAP-1-mediated intracellular actions. Both CRISPR-based KOs of LPHN-1 significantly reduced the TCAP-1 associated increase in intracellular Ca^2+^. We were unsuccessful to create a LPHN-3 KO however. The length of the LPHN-3 gene in mice is about 10 times that of the LPHN-1 gene due to much longer intronic sequences. Thus, this extended sequence may have played a role in the lack of viability of the CRISPR-associated LPHN-3 oligonucleotides. Moreover, TCAP-1-mediated intracellular Ca^2+^ was established using pharmacological antagonists of the PLC-IP3-IP3R pathway of the SR. This rise in intracellular Ca^2+^ led to increased cellular glucose, concomitant with increases in ATP and NADH production. Indeed, the TCAP-1-mediated increase in intracellular Ca^2+^ corroborated with MT membrane hyperpolarization indicating that TCAP-1 also acted to increase MT activity. However, the siRNA oligonucleotides inhibited the actions of both receptors as indicated by the significant reduction in the TCAP-1-mediated cytosolic Ca^2+^ response. SiRNA KDs of the LPHN-1 and -3 has been successfully used in the past using the mouse pancreatic β-cell line, MIN6 Röthe et al., 2019 where the authors showed that LPHN-3 KDs reduced insulin secretion by reducing cAMP levels *via* a Gi-mediated pathway. Although we have not examined the direct role of TCAP-1 on pancreatic insulin release, this study is consistent with our supposition that TCAP, itself, is associated, in part, with glucose regulation *in vivo* (Hogg et al., 2018; Lovejoy and Hogg, 2020). In our current study, however, our experiments showed that ablation of LPHN-1 or -3 reduced TCAP-1-mediated Ca^2+^ concentrations toward baseline levels. These results surprised us. We expected that the KO or KD or either receptor would render only a partial suppression of the Ca^2+^ response. Because this was not the case, one possibility is that there is an interaction among the LPHN isoforms specifically, or their combined actions with the teneurins.

Teneurin and LPHN interaction is complex where the stoichiometry among the 4 teneurin and three LPHN paralogs has not been ascertained. Although studies using vertebrate models establish evidence of teneurin-LPHN interaction (Silva et al., 2011; Jackson et al., 2018; Li et al., 2018; Araç and Li, 2019), the specific correspondence of any teneurin with any LPHN as cognitive pairs has yet to be established. The teneurins are multifunctional transmembrane proteins that have TCAP at their distal extracellular tip. Even less is understood regarding the promiscuity of the LPHNs with respect to TCAP interactions. Previously, Silva and his associates (Silva et al., 2011) showed that the teneurin-2 region possessing the TCAP unit was required for full binding to the LPHN-1 (Lasso), and Husic and her colleagues (Husic et al., 2019) showed that the transgenic expression of teneurin-1 TCAP co-precipitated with the transgenic over-expressed hormone-binding domain (HBD) of LPHN-1 and, moreover, modulated the cell adhesion characteristics of HEK293 cells over-expressed with the LPHN-1 mRNA. In this current study, we established that both LPHN-1 and teneurin-3 mRNA transcripts were present and their immunoreactive associated epitopes are co-localized in the sarcolemma (Figure 3). Although these data corroborate with the PCR basal mRNA expression data indicating that these specified mRNA transcripts show the highest expression, the data are not meant to suggest that this is indicative of cognitive ligand and receptor pairs, *per se.*

There is evidence that LPHN paralogs interact with each other. In our LPHN-1 and -3 attenuation studies, the reduction of one receptor inhibited the TCAP-1 mediated Ca^2+^ actions of the other receptor. It is possible that there is a minor Ca^2+^ response mediated by the non-target LPHN, but too low to detect with our assay conditions, although this seems unlikely. Homo-and heterophilic oligomerization is a characteristic of the GPCRs, but this has not been well-studied in Adhesion GPCR family members (Meza-Aguilar and Boucard, 2014; Milligan et al., 2019). In LPHN-1, the C-terminal fragment and N-terminal fragment is cleaved *in vivo* and re-associates with the α-latrotoxin (αLTX) mutant ligand LTX^N4C^ (Volynski et al., 2004), although this phenomenon has not been studied in detail in other LPHN paralogs despite the high degree of conservation among these domains. Further, studies using the ‘*stachel*’ peptide have provided additional insight into potential LPHN paralog interactions with each other (Mϋller et al., 2015; Nazarko et al., 2015; Röthe et al., 2019). The *stachel* peptide represents the N-terminus of the C-terminal fragment following cleavage of the conserved GPCR-autoproteolysis (GAIN) region (Liebscher et al., 2014). The recombinantly expressed *stachel* peptide induces diverse G-protein associations across LPHN paralogs, whereas αLTX favours G11 signalling *via* LPHN-1 (Lelianova et al., 1997; Davletov et al., 1998; Rahman et al., 1999). We and others have previously shown that the TCAP amino acid sequence resembles that of Secretin GPCR family ligands and αLTX (Michalec et al., 2020), thus we posit that TCAP may represent the endogenous ligand that αLTX co-evolved with to become a toxin.

There were questions we did not address in our study. One such question were the triggers that stimulate TCAP to regulate skeletal muscle physiology. Our hypothesis, at this time, is that TCAP is liberated locally in the sarcolemma either by a direct cleavage of the teneurin (Lovejoy et al., 2006; Jackson et al., 2018; Li et al., 2018) or by independent mRNA transcription, translation and release from skeletal muscle or other local tissues (Chand et al., 2013b; Reid et al., 2021). We have shown in the past that TCAP-1 can increase teneurin transcription in immortalized neurons (Wang et al., 2005), but we have yet to establish this *in vivo*. Although TCAP is highly expressed in the brain, our studies showing its presence in as a circulating hormone in serum has been equivocal (Lovejoy, unpublished observations). As this is a critical aspect of TCAP action, this is a goal for upcoming studies. Another questions we did not resolve was the role of cellular stress with respect to TCAP-1 action on skeletal muscle cells and its role in sarcolemma and mitochondrial polarization dynamics. In the current study, we used only non-stressed conditions to understand the role of TCAP-1 on skeletal muscle cells. Further studies will need to be performed to establish the role of TCAP under different metabolic and stress-associated conditions.

In summary, our data in this study indicate that TCAP-1 regulates energy metabolism in skeletal muscle *via* an insulin-independent mechanism, and by doing so, modulates contractile kinetics, *via* Ca^2+^ dynamics and ATP production. Together, these data describe a previously undescribed mechanism to regulate skeletal muscle dynamics. These data provide the foundation for a proposed mechanism of TCAP-1 action in skeletal muscle. As a hypothesis, we suggest that TCAP-1 interacts with LPHN-1 and -3 to stimulate the activation of G-protein-coupled PLC leading to the increased conversion of PIP3 into IP3 and DAG. Increased IP3 levels stimulates the IP3R on the SR, opening Ca^2+^ channels to increase cytosolic Ca^2+^ levels. Cytosolic Ca^2+^ is imported into the MT likely *via* the Ca^2+^ uniporter (MCU) which stimulates the TCA cycle and ETC. Enhanced ETC activity results in increased proton extrusion from the MT matrix and hyperpolarization of MT membrane potential. This ultimately results in increased ATP and NADH production, as well as increased ATP levels. Ca^2+^ is subsequently pumped out of the MT likely *via* Na^+^/Ca^2+^ exchangers (NCX), thus restoring homeostatic levels of Ca^2+^. Moreover, we showed that the TCAP-1 increased cellular energy availability by increased glucose importation into cells likely due to increased GLUT4 expression. The TCAP-1 mediated mechanism is likely due to its interactions with LPHNs -1 and -3.

## Data Availability

The original contributions presented in the study are included in the article/supplementary materials, further inquiries can be directed to the corresponding author.

## References

[B1] Al Chawaf A. Amant K. S. Belsham D. D. Lovejoy D. A. (2007). Regulation of neurite growth in immortalized mouse hypothalamic neurons and rat hippocampal primary cultures by teneurin C-terminal-associated peptide-1. Neuroscience 144, 1241–1254. 10.1016/j.neuroscience.2006.09.062 17174479

[B2] Al Chawaf A. Xu K. Tan L. Vaccarino F. Lovejoy D. A. Rotzinger S. (2007). Corticotropin-releasing factor (CRF)-induced behaviors are modulated by intravenous administration of teneurin C-terminal associated peptide-1 (TCAP-1). Peptides 28, 1406–1415. 10.1016/j.peptides.2007.05.014 17644218

[B3] Araç D. Li J. (2019). Teneurins and latrophilins: Two giants meet at the synapse. Curr. Opin. Struct. Biol. 54, 141–151. 10.1016/j.sbi.2019.01.028 30952063 PMC6677252

[B4] Aravind L. Anantharaman V. Zhang D. de Sousa R. F. Iyer L. M. (2012). Gene flow and biological conflict systems in the origin and evolution of eukaryotes. Front. Cell. Infect. Microbiol. 2, 89. 10.3389/fcimb.2012.00089 22919680 PMC3417536

[B5] Baumgartner S. Martin D. Hagios C. Chiquet-Ehrismann R. (1994). Tenm, a Drosophila gene related to tenascin, is a new pair-rule gene. EMBO J. 13, 3728–3740. 10.1002/j.1460-2075.1994.tb06682.x 8070401 PMC395283

[B6] Calderón J. C. Bolaños P. Caputo C. (2014). The excitation–contraction coupling mechanism in skeletal muscle. Biophys. Rev. 6, 133–160. 10.1007/s12551-013-0135-x 28509964 PMC5425715

[B7] Chand D. Casatti C. A. De Lannoy L. Song L. Kollara A. Barsyte-Lovejoy D. (2013). C-terminal processing of the teneurin proteins: Independent actions of a teneurin C-terminal associated peptide in hippocampal cells. Mol. Cell. Neurosci. 52, 38–50. 10.1016/j.mcn.2012.09.006 23026563

[B8] Chand D. De Lannoy L. Tucker R. P. Lovejoy D. A. (2013). Origin of chordate peptides by horizontal protozoan gene transfer in early metazoans and protists: Evolution of the teneurin C-terminal associated peptides (TCAP). Gen. Comp. Endocrinol. 188, 144–150. 10.1016/j.ygcen.2013.02.006 23453965

[B9] Chand D. Song L. De Lannoy L. Barsyte-Lovejoy D. Ackloo S. Boutros P. C., (2012). C-Terminal region of teneurin-1 co-localizes with dystroglycan and modulates cytoskeletal organization through an extracellular signal-regulated kinase-dependent stathmin- and filamin A-mediated mechanism in hippocampal cells. Neuroscience 219, 255–270. 10.1016/j.neuroscience.2012.05.069 22698694

[B10] Davletov B. A. Meunier F. A. Ashton A. C. Matsushita H. Hirst W. D. Lelianova V. G., (1998). Vesicle exocytosis stimulated by alpha-latrotoxin is mediated by latrophilin and requires both external and stored Ca^2+^ . EMBO J. 17, 3909–3920. 10.1093/emboj/17.14.3909 9670008 PMC1170726

[B11] Davletov B. A. Shamotienko O. G. Lelianova V. G. Grishin V. Ushkaryov Y. A. (1996). Isolation and biochemical characterization of a Ca2+-independent alpha-latrotoxin-binding protein. J. Biol. Chem. 1271, 23239–23245. 10.1074/jbc.271.38.23239 8798521

[B12] Denton R. M. (2009). Regulation of mitochondrial dehydrogenases by calcium ions. Biochim. Biophys. Acta 1787, 1309–1316. 10.1016/j.bbabio.2009.01.005 19413950

[B13] Dias-Vegas A. R. Cardova A. Valladares D. Llanos P. Hildago C. Gharardi G. (2018). Mitochondrial calcium increase induced by RyR1 and IP_3_R channel activation after membrane polarizations regulates skeletal muscle metabolism. Front. Physiol. 9, 791–799. 10.3389/fphys.2018.00791 29988564 PMC6026899

[B14] Erb S. McPhee M. Brown Z. J. Kupferschmidt D. A. Song L. Lovejoy D. A. (2014). Repeated intravenous administrations of teneurin-C terminal associated peptide (TCAP)-1 attenuates reinstatement of cocaine seeking by corticotropin-releasing factor (CRF) in rats. Behav. Brain Res. 269, 1–5. 10.1016/j.bbr.2014.04.013 24768621

[B15] Fredricksson R. Lagerstrom L. Lundin L. G. Schioth H. (2003). The G protein coupled receptors in the human genome form five main families. Phylogenetic analysis, paralogon groups and fingerprints. Mol. Pharmacol. 63, 1256–1272. 10.1124/mol.63.6.1256 12761335

[B16] Fredriksson R. Schiöth H. (2005). The repertoire of G-protein coupled receptors in fully sequenced genomes. Mol. Pharmacol. 67, 1414–1425. 10.1124/mol.104.009001 15687224

[B17] Hogg D. W. Chen Y. D’Aquila A. L. Xu M. Husic M. Tan L. A., (2018). A novel role of the corticotrophin-releasing hormone regulating peptide, teneurin C-terminal associated peptide 1, on glucose uptake into the brain. J. Neuroendocrinol. 30, e12579. 10.1111/jne.12579 29411913

[B18] Holwerda A. M. Locke M. (2014). Hsp25 and Hsp72 content in rat skeletal muscle following controlled shortening and lengthening contractions. Appl. Physiol. Nutr. Met. 39, 1380–1387. 10.1139/apnm-2014-0118 25356915

[B19] Hong W. Mosca T. J. Luo L. (2012). Teneurins instruct synaptic partner matching in an olfactory map. Nature 484, 201–207. 10.1038/nature10926 22425994 PMC3345284

[B20] Husic M. Barsyte-Lovejoy D. Lovejoy D. A. (2019). Teneurin C-terminal associated peptide (TCAP)-1 and latrophilin interaction in HEK293 cells: Evidence for modulation of intercellular adhesion. Front. Endocrinol. 10, 22. 10.3389/fendo.2019.00022 PMC6367273 30774623

[B21] Ishii K. Suzuki N. Mabuchi Y. Ito N. Kikura N. Fukada S. I., (2015). Muscle satellite cell protein teneurin-4 regulates differentiation during muscle regeneration. Stem Cells 33, 3017–3027. 10.1002/stem.2058 26013034 PMC4744701

[B22] Jackson V. A. Meijer D. H. Carrasquero M. van Bezouwen L. S. Lowe E. D. Kleanthous C., (2018). Structures of Teneurin adhesion receptors reveal an ancient fold for cell-cell interaction. Nat. Commun. 9, 1079. 10.1038/s41467-018-03460-0 29540701 PMC5851990

[B23] Kenzelmann D. Chiquet-Ehrismann R. Leachman N. T. Tucker R. P. (2008). Teneurin-1 is expressed in interconnected regions of the developing brain and is processed *in vivo* . BMC Dev. Biol. 8, 30. 10.1186/1471-213X-8-30 18366734 PMC2289808

[B24] King N. Hittinger C. T. Carroll S. B. (2003). Evolution of key cell signaling and adhesion protein families predates animal origins. Science 301, 361–363. 10.1126/science.1083853 12869759

[B25] King N. Westbrook M. J. Young S. L. Kuo A. Abedin M. Chapman J., (2008). The genome of the choanoflagellate Monosiga brevicollis and the origin of metazoans. Nature 451, 783–788. 10.1038/nature06617 18273011 PMC2562698

[B26] Kupferschmidt D. Lovejoy D. A. Rotzinger S. Erb S. (2011). Teneurin C-terminal associated peptide-1 blocks the effects of corticotropin-releasing factor on reinstatement of cocaine seeking and on cocaine-induced behavioural sensitization. Br. J. Pharmacol. 163, 574–583. 10.1111/j.1476-5381.2010.01055.x PMC3041248 20883474

[B27] Lang J. Ushkaryov Y. Grasso A. Wollheim C. B. (1998). Ca2+-independent insulin exocytosis induced by alpha-latrotoxin requires latrophilin, a G protein-coupled receptor. EMBO J. 17, 648–657. 10.1093/emboj/17.3.648 9450990 PMC1170414

[B28] Lelianova V. G. Davletov B. A. Sterling A. Atiqur Rahman M. Grishin E. V. Totty N. F., (1997). Alpha-latrotoxin receptor, latrophilin, is a novel member of the secretin family of G protein-coupled receptors. J. Biol. Chem. 272, 21504–21508. 10.1074/jbc.272.34.21504 9261169

[B29] Levine A. Bashan-Ahrend A. Budai-Hadrian O. Gartenber D. Menasherow S. Wides R. (1994). Odd oz: A novel Drosophila pair-rule gene. Cell 77, 587–598. 10.1016/0092-8674(94)90220-8 7514504

[B30] Li J. Shalev-Benami M. Sando R. Jiang X. Kibrom A. Wang J., (2018). Structural basis for teneurin function in circuit-wiring: A toxin motif at the synapse. Cell 173, 735–748. 10.1016/j.cell.2018.03.036 29677516 PMC5912346

[B31] Liang H. Ward W. F. (2006). PGC-1alpha: A key regulator of energy metabolism. Adv. Physiol. Educ. 30, 145–151. 10.1152/advan.00052.2006 17108241

[B32] Liebscher I. Schön J. Peterson S. C. Fischer L. Auerback N. Demberg L. M., (2014). A tethered agonist within the ectodomain activates the adhesion G protein-coupled receptors GPR126 and GPR133. Cell Rep. 9, 2018–2026. 10.1016/j.celrep.2014.11.036 25533341 PMC4277498

[B33] Lovejoy D. A. Al Chawaf A. Cadinouche A. (2006). Teneurin C-terminal associated peptides: An enigmatic family of neuropeptides with structural similarity to the corticotropin-releasing factor and calcitonin families of peptides. Gen. Comp. Endocrinol. 148, 299–305. 10.1016/j.ygcen.2006.01.012 16524574

[B34] Lovejoy D. A. Hogg D. W. (2020). Information processing in affective disorders: Did an ancient peptide regulating intercellular metabolism become co-opted for noxious stress sensing? BioEssays, 42, 2000039. 10.1002/bies.202000039 32767437

[B35] Maher F. (1995). Immunolocalization of GLUT1 and GLUT3 glucose transporters in primary cultured neurons and glia. J. Neurosci. Res. 42, 459–469. 10.1002/jnr.490420404 8568932

[B36] Meza-Aguilar D. G. Boucard A. A. (2014). Latrophilins updated. Biomol. Concepts 5, 457–478. 10.1515/bmc-2014-0032 25429599

[B37] Michalec O. M. Chang B. Lovejoy N. Lovejoy D. A. (2020). Corticotropin-releasing factor: An ancient peptide family related to the secretin peptide superfamily. Front. Endocrinol. 11, 529. 10.3389/fendo.2020.00529 PMC7481443 32973673

[B38] Milligan G. Ward R. J. Marsango S. (2019). GPCR homo-oligomerization. Curr. Opin. Cell Biol. 57, 40–47. 10.1016/j.ceb.2018.10.007 30453145 PMC7083226

[B39] Minet A. D. Chiquet-Ehrismann R. (2000). Phylogenetic analysis of teneurin genes and comparison to the rearrangement hotspot elements of *E. coli* . Gene 257, 87–97. 10.1016/s0378-1119(00)00388-7 11054571

[B40] Minet A. D. Rubin B. P. Tucker R. P. Baumgartner S. Chiquet-Ehrismann R. (1999). Teneurin-1, a vertebrate homologue of the Drosophila pair-rule gene ten-m, is a neuronal protein with a novel type of heparin-binding domain. J. Cell Sci. 112, 2019–2032. 10.1242/jcs.112.12.2019 10341219

[B41] Mosca T. J. Hong W. Dani V. S. Favaloro V. Luo L. (2012). Trans-synaptic Teneurin signalling in neuromuscular synapse organization and target choice. Nature 484, 237–241. 10.1038/nature10923 22426000 PMC3326183

[B42] Mϋller A. Winkler J. Fiedler F. Sastraihardja T. Binder C. Schnabel R., (2015). Oriented cell division in the *C. elegans* embryo is coordinated by G-protein signaling dependent on the adhesion GPCR LAT-1. PLoS Genet. 11, e1005624. 10.1371/journal.pgen.1005624 26505631 PMC4624771

[B43] Nazarko O. Kibrom A. Winkler J. Leon K. Stoveken H. Salzman G., (2015). A comprehensive mutagenesis screen of the adhesion GPCR latrophilin-1/ADGRL1. iScience 3, 264–278. 10.1016/j.isci.2018.04.019 PMC6137404 30428326

[B44] Ni T. Yue J. Sun G. Zou Y. Wen J. Huang J. (2012). Ancient gene transfer from algae to animals: Mechanisms and evolutionary significance. BMC Evol. Biol. 12, 83. 10.1186/1471-2148-12-83 22690978 PMC3494510

[B45] Oohashi T. Zhou X. H. Feng K. Richter B. Mörgelin M. Perez M. T., (1999). Mouse ten-m/Odz is a new family of dimeric type II transmembrane proteins expressed in many tissues. J. Cell Biol. 145, 563–577. 10.1083/jcb.145.3.563 10225957 PMC2185078

[B46] Qian X. Barsyte-Lovejoy D. Chewpoy R. B. Wang L. Gautam N. Wang N., (2004). Cloning and characterization of teneurin C-terminus associated peptide (TCAP)-3 from the hypothalamus of an adult rainbow trout (*Oncorhynchus mykiss*). Gen. Comp. Endocrinol. 137, 205–216. 10.1016/j.ygcen.2004.02.007 15158132

[B47] Rahman M. A. Ashton A. C. Meunier F. Davletov B. A. Dolly J. O. Ushkaryov Y. A. (1999). Norepinephrine exocytosis stimulated by alpha-latrotoxin requires both external and stored Ca2+ and is mediated by latrophilin, G proteins and phospholipase C. Philos. Trans. R. Soc. Lond. B Biol. Sci. 354, 379–386. 10.1098/rstb.1999.0390 10212487 PMC1692485

[B48] Ramulu H. G. Raoult D. Pontarotti P. (2012). The rhizome of life: What about metazoans? Cell. Infect. Microbiol. 2, 1–11. 10.3389/fcimb.2012.00050 PMC3417402 22919641

[B49] Reid R. M. Reid A. L. Lovejoy D. A. Biga P. R. (2021). Teneurin C-terminal associated peptide (TCAP)-3 increases metabolic activity in zebrafish. Front. Mar. Sci. 7, 591160. 10.3389/fmars.2020.591160

[B50] Richter E. A. Hargraves M. (2013). Exercise, GLUT4, and skeletal muscle glucose uptake. Physiol. Rev. 93, 993–1017. 10.1152/physrev.00038.2012 23899560

[B51] Rolfe D. F. Brand M. D. (1997). The physiological significance of mitochondrial proton leak in animal cells and tissues. Biosci. Rep. 17, 9–16. 10.1023/a:1027327015957 9171916

[B52] Röthe J. Thor R. Winkler J. Knierim A. B. Binder C. Huth S., (2019). Involvement of the adhesion GPCRs latrophilins in the regulation of insulin release. Cell Rep. 26, 1573–1584. 10.1016/j.celrep.2019.01.040 30726739

[B53] Rotzinger S. Lovejoy D. A. Tan L. (2010). Behavioral effects of neuropeptides in rodent models of depression and anxiety. Peptides 31, 736–756. 10.1016/j.peptides.2009.12.015 20026211

[B54] Rubin B. P. Tucker R. P. Martin D. Chiquet-Ehrismann R. (1999). Teneurins: A novel family of neuronal cell surface proteins invertebrates, homologous to the Drosophila pair-rule gene product ten-m. Dev. Biol. 216, 195–209. 10.1006/dbio.1999.9503 10588872

[B55] Sando R. Südhof T. C. (2021). Latrophilin GPCR signaling mediates synapse formation. Elife 10, e65717. 10.7554/eLife.65717 33646123 PMC7954527

[B56] Santos J. M. Ribeiro S. B. Gaya A. R. Appell H. J. Duarte J. A. (2008). Skeletal muscle pathways of contraction-enhanced glucose uptake. Int. J. Sports Med. 29, 785–794. 10.1055/s-2008-1038404 18401805

[B57] Sayer A. A. Dennison E. M. Syddall H. E. Gilbody H. J. Philips D. I. W. Cooper C. (2005). Type 2 diabetes, muscle strength, and impaired physical function: The tip of the iceberg? Diabetes Care 28, 2541–2542. 10.2337/diacare.28.10.2541 16186295

[B58] Sekar R. Chow B. K. C. (2013). Role of secretin peptide family and their receptors in the hypothalamic control of energy homeostasis. Horm. Metab. Res. 45, 945–954. 10.1055/s-0033-1353155 24068610

[B59] Silva J.-P. Ushkaryov Y. A. (2010). The latrophilins, “split-personality” receptors. Adv. Exp. Med. Biol. 706, 59–75. 10.1007/978-1-4419-7913-1_5 21618826 PMC3145135

[B60] Silva J. P. Lelianova V. G. Ermolyuk Y. S. Vysokov N. Hitchen P. G. Berninghausen O., (2011). Latrophilin 1 and its endogenous ligand Lasso/teneurin-2 form a high-affinity trans-synaptic receptor pair with signaling capabilities. Proc. Natl. Acad. Sci. U. S. A. 108, 12113–12118. 10.1073/pnas.1019434108 21724987 PMC3141932

[B61] Strotmann R. Schrock K. Boselt I. Staubert C. Russ A. Schoneberg T. (2011). Evolution of GPCR: Change and continuity. Mol. Cell. Endocrinol. 331, 170–178. 10.1016/j.mce.2010.07.012 20708652

[B62] Tan L. A. Al Chawaf A. Vaccarino F. J. Boutros J. C. Lovejoy D. A. (2011). Teneurin C-terminal associated peptide (TCAP)-1 modulates dendritic morphology in hippocampal neurons and decreases anxiety-like behaviors in rats. Physiol. Behav. 104, 199–204. 10.1016/j.physbeh.2011.03.015 21411044

[B63] Tan L. A. Chand D. De Almeida R. Xu M. Colacci M. De Lannoy L., (2012). Modulation of neuroplastic changes and corticotropin-releasing factor-associated behavior by a phylogenetically ancient and conserved peptide family. Gen. Comp. Endocrinol. 176, 309–313. 10.1016/j.ygcen.2011.11.011 22138219

[B64] Tan L. Xu K. Vaccarino F. Lovejoy D. A. Rotzinger S. (2008). Repeated intracerebral teneurin C-terminal associated peptide (TCAP)-1 injections produce enduring changes in behavioral responses to corticotropin-releasing factor (CRF) in rat models of anxiety. Behav. Brain Res. 188, 195–200. 10.1016/j.bbr.2007.10.032 18082275

[B65] Tucker R. P. Chiquet-Ehrismann R. (2006). Teneurins: A conserved family of transmembrane proteins involve in intercellular signaling during development. Dev. Biol. 290, 237–245. 10.1016/j.ydbio.2005.11.038 16406038

[B66] Tucker R. P. (2013). Horizontal gene transfer in choanoflagellates. J. Exp. Zool. B Mol. Dev. Evol. 320, 1–9. 10.1002/jez.b.22480 22997182

[B67] Uemura E. Greenlee H. W. (2006). Insulin regulates neuronal glucose uptake by promoting translocation of glucose transporter GLUT3. Exp. Neurol. 198, 48–53. 10.1016/j.expneurol.2005.10.035 16337941

[B68] Volynski K. A. Silva J.-P. Lelianova V. G. Atiqur Rahman M. Hopkins C. Ushkaryov Y. A. (2004). Latrophilin fragments behave as independent proteins that associate and signal on binding of LTX(N4C). EMBO J. 23, 4423–4433. 10.1038/sj.emboj.7600443 15483624 PMC526461

[B69] Wan B. La Noue K. F. Cheung J. Y. Scaduto R. C. (1989). Regulation of citric acid cycle by calcium. J. Biol. Chem. 264, 13430–13439. 10.1016/s0021-9258(18)80015-1 2503501

[B70] Wang L. Rotzinger S. Barsyte-Lovejoy D. Qian X. Elias C. F. Bittencourt J. C., (2005). Teneurin proteins possess a carboxy terminal corticotropin-releasing factor-like sequence that modulates emotionality and neuronal growth. Mol. Brain Res. 133, 253–265. 15710242 10.1016/j.molbrainres.2004.10.019

[B71] Young T. R. Leamey C. A. (2009). Teneurins: Important regulators of neural circuitry. Int. J. Biochem. Cell Biol. 41, 990–993. 10.1016/j.biocel.2008.06.014 18723111

[B72] Zhang D. de Souza R. F. Anantharaman V. Iyer L. M. Aravind L. (2012). Polymorphic toxin systems: Comprehensive characterization of trafficking modes, processing, mechanisms of action, immunity and ecology using comparative genomics. Biol. Direct 7, 18. 10.1186/1745-6150-7-18 22731697 PMC3482391

